# Removal of Pharmaceutical Residues from Water and Wastewater Using Dielectric Barrier Discharge Methods—A Review

**DOI:** 10.3390/ijerph18041683

**Published:** 2021-02-10

**Authors:** Emile S. Massima Mouele, Jimoh O. Tijani, Kassim O. Badmus, Omoniyi Pereao, Omotola Babajide, Cheng Zhang, Tao Shao, Eduard Sosnin, Victor Tarasenko, Ojo O. Fatoba, Katri Laatikainen, Leslie F. Petrik

**Affiliations:** 1 Environmental Nano Science Research Group, Department of Chemistry, University of the Western Cape, Bellville, Cape Town 7535, South Africa; 3282790@myuwc.ac.za (J.O.T.); 3481395@myuwc.ac.za (K.O.B.); pereaokola@gmail.com (O.P.); Omotola.babajide@gmail.com (O.B.); larryfat2006@gmail.com (O.O.F.); 2 Department of Separation Science, Lappeenranta-Lahti University of Technology LUT, P.O. Box 20, FI-53851 Lappeenranta, Finland; Katri.Laatikainen@lut.fi; 3 Department of Chemistry, Federal University of Technology, PMB 65, P.O. Box 920 Minna, Niger State 920001, Nigeria; 4 Department of Mechanical Engineering, Cape Peninsula University of Technology, P.O. Box 1906, Bellville 7535, South Africa; 5 Beijing International S&T Cooperation Base for Plasma Science, Energy Conversion, Institute of Electrical Engineering, Chinese Academy of Sciences, Beijing 100190, China; zhangcheng@mail.iee.ac.cn (C.Z.); st@mail.iee.ac.cn (T.S.); 6 Institute of High Current Electronics, Russian Academy of Sciences, 634055 Tomsk, Russia; badik@loi.hcei.tsc.ru (E.S.); vft@loi.hcei.tsc.ru (V.T.)

**Keywords:** pharmaceutical residues, water, wastewater, dielectric barrier discharge, advanced oxidation technologies, chemicals/contaminants of emerging concern (CEC), excilamp

## Abstract

Persistent pharmaceutical pollutants (PPPs) have been identified as potential endocrine disruptors that mimic growth hormones when consumed at nanogram per litre to microgram per litre concentrations. Their occurrence in potable water remains a great threat to human health. Different conventional technologies developed for their removal from wastewater have failed to achieve complete mineralisation. Advanced oxidation technologies such as dielectric barrier discharges (DBDs) based on free radical mechanisms have been identified to completely decompose PPPs. Due to the existence of pharmaceuticals as mixtures in wastewater and the recalcitrance of their degradation intermediate by-products, no single advanced oxidation technology has been able to eliminate pharmaceutical xenobiotics. This review paper provides an update on the sources, occurrence, and types of pharmaceuticals in wastewater by emphasising different DBD configurations previously and currently utilised for pharmaceuticals degradation under different experimental conditions. The performance of the DBD geometries was evaluated considering various factors including treatment time, initial concentration, half-life time, degradation efficiency and the energy yield (G_50_) required to degrade half of the pollutant concentration. The review showed that the efficacy of the DBD systems on the removal of pharmaceutical compounds depends not only on these parameters but also on the nature/type of the pollutant.

## 1. Introduction

The availability of clean water is fundamental to the socio-economic development and maintenance of human health. Surface water has become a source of considerable environmental concern due to growing discharge and detection of chemicals of emerging concern at nanogram or microgram per litre levels [1,2,3,4,5,6]. Chemicals of emerging concern (CEC) have been explicitly defined by the United States Geological Survey [7] as any synthetic or naturally occurring chemical or microbial constituent previously not detected or historically known or considered to be a contaminant, but which interferes with hormonal functions in the body. CEC may also include substances that have long been in the environment but whose occurrence and importance are recently being elucidated as a consequence of advanced analytical techniques [8]. Emerging micropollutants include pharmaceuticals, endocrine disruptors, flame retardants, nanomaterials and perfluorinated compounds [9,10,11]. An article by Roh et al. [12] reported swallowing a drug for medical remediation had anticipated effects in the body, although the final destination of these substances was not considered due to incomplete metabolism, part of the drugs may be excreted in urine or faeces may eventually end up in wastewater treatment plants [13]. Pharmaceuticals and their metabolites get released into aquatic environments through land-application of sewage sludge effluents from treatment plants, as well as surface water runoff from industries. A growing concern among water and wastewater treatment industries about the presence of xenobiotics in water resources and their removal from water has advocated for effective water treatment. It is important to mention that the occurrence of pharmaceuticals in the environment has become pervasive, presenting a life-threatening issue that affects humans and ecosystems [14]. The concentration of pharmaceuticals and pharmaceutical residues in the environment varies and depends largely on industrial activities, human consumption patterns, wastewater treatment capacity, population growth among others [15]. Due to high levels of apprehension concerning the recent findings of the toxicity of pharmaceuticals at low concentrations, their removal has become a matter of urgency to the water industry and other regulatory agencies. Some physical, chemical and biological approaches such as filtration, adsorption, precipitation, coagulation and flocculation have been utilised for wastewater treatment [16,17,18,19,20]. Microbial electrochemical techniques (MET) are extended remediation techniques that employ Microbial Fuel Cells (MFCs) or Microbial Electrolysis cells (MECs) and focus on the same chemical principles as those of coagulation, flotation, Fenton oxidation, chemical oxidation, and adsorption [21,22]. These systems generate hydroxyl radicals and various co-species including sulphates and hydrogen carbonates that contribute to the removal of persistent organic pollutants [23]. The MFCs and MECs function using microbes to extract electrical power from the organic constituents, and hence rendering MET energy-autonomous and promising methods for the future [24]. However, the diversity of MET aiming at different microbial cultures and practical applications is a challenge in selecting/defining which method is precise and suitable for specific research query. To comply with stringent environmental regulatory frameworks and to reduce the pressure on existing limited clean water, alternative treatment techniques based on advanced oxidation processes (AOPs) need to be developed [10,25]. These AOPs are viable technologies capable of decomposing biologically recalcitrant and persistent organic pollutants present in wastewater [26,27], easily accomplished using hydroxyl radicals as primary non-selective oxidants [28]. The common AOPs reported in the literature include UV disinfection (photolysis) and various photolytic combinations including UV/H_2_O_2_, UV/O_3_, UV/H_2_O_2_/O_3_ photocatalysis, electron-beam, plasma technologies, supercritical water oxidation, wet air oxidation, water sonolysis, ultrasound cavitation, electrochemical advanced oxidation processes (EAOPs), microwave plasma-based AOPs [20,29,30,31]. Although these procedures use different systems, they all converge towards the production of powerful but short-lived non-toxic and non-selective hydroxyls radical (OH^.^) and other oxidative and reducing species such as H^.^, O^.^, O_2_^.−^, H_2_O_2_, etc. that mineralize water contaminants into simpler by-products. Various AOPs have been employed to oxidize and mineralise organic pollutants such as dyes, pharmaceuticals, and personal care product into CO_2_, water, and harmless organic and inorganic species [4,11,25,32,33,34,35,36,37,38]. In most AOPs, series of gaseous, liquid chemicals including O_3_, H_2_O_2_, catalysts, and external UV sources have been used and the total decomposition of pollutants often requires extended treatment time. These in return involve a lot of wet chemistry and some have an increase in the operational costs [39,40,41,42]. Alternatively, non-thermal electrical discharge systems have been found very effective due to the generation of different molecular and ionic free reactive species, UV radiation, and shock waves at ambient conditions without chemical additives [43]. The combination of these species forms a mixture of potent oxidants in the bulk solution that mineralises water pollutants in a short period [44,45,46,47]. AOPs induced by dielectric barrier discharge (DBD) are considered more effective due to their plasma properties and reactor configurations [47,48,49]. The prominence of DBDs over other configurations relies mostly on the electron energy fluctuating between 1 and 10 eV and the electron density that varies between 10^12^ and 10^15^ cm^−3^. DBD systems require less or no chemical additives, regardless of the feed gas such as air, oxygen, or argon, 1 eV is enough to produce stable UV-light, shock waves, and various free molecular and radical species including O_3_, H_2_O_2_, O**^.^**, O_2_^.−^, OH**^.^**, etc. [20]. The mixture of these oxidants represents a powerful chemical cocktail that induces complete removal of water contaminants [4,50]. The decomposition of organic toxins from water using different DBD configurations with total degradation achieved in reduced plasma exposure times has recently been reported [51,52,53,54]. These investigations clearly show the supremacy of DBD technologies over co-existing AOPs. This review highlights the abundance of various classes of pharmaceuticals in water sources and focuses on their removal by different DBD systems and configurations.

## 2. Pharmaceutical Residues in Water and Wastewater

The profusion of pharmaceutical residues in aquatic environments has triggered worldwide environmental research recently, though the appearance and accumulation of these xenobiotics in water streams are not new, their accumulation in water sources and water treatment facilities has increasingly been recounted. The presence of these toxins in water has a globally limited water supply, leading to potable/drinking water. Indeed, the accumulation of pharmaceutical compounds (PhCs) in water and wastewater treatment plants has rendered the treated effluents non-reusable since the sludge produced is toxic and highly contaminated with PhCs. The contaminants present in treated effluents show that they could not be removed by conventional treatment methods and are often discharged into the environments without being treated [2,4,5]. The world’s consumption of pharmaceuticals could be estimated in hundreds of tonnes (t). For instance, in Germany, millions of non-steroidal anti-inflammatory drugs (NSAIDS) such as aspirin, paracetamol, ibuprofen, and diclofenac were produced during 2000 and 2001 corresponding to 86 (t) [55]. Besides Germany, France and Switzerland are also giant consumers of pharmaceutical products (PPs) in Europe [56]. The abundance of PhCs in aquatic environments vary from one country to another and may depend on the practice of prescription methods [57,58,59]. In Italy, Ferrari et al. [60] investigated the accumulation of pharmaceuticals in surface water and sediments in the largest Italian River Po basin. Out of the 50 samples taken from the sources to the delta and the mouth of its major effluents, the following trace pharmaceuticals: atenolol, propranolol, carbamazepine, ranitidine, metronidazole, paracetamol and atorva were detected in the concentration range of 0.38–3.59 µg/L. The authors claimed that though such concentrations may be disrupting to aquatic ecosystems, they present a low risk to human beings. Ayman and Isik [61] studied the occurrence of various (nine) pharmaceutical active compounds (PACs) in hospital wastewater, sewage wastewater, drinking water and treated water. Results showed that acetaminophen and caffeine were the most frequently detected compounds with concentrations up to 160 µg/L in both hospital and sewage wastewater although no traces of these contaminants were found in drinking water.

A similar study on environmental monitoring was carried out by Rodivic et al. [62] that developed a practical solid-phase extraction analytical method coupled with GC-MS for the detection of PhCs in river sediments and corresponding surface and groundwater in the Dambe River and tributaries in Serbia. The research outcome revealed that the pesticides dimethoate and atrazine were the frequently detected PACs in sediments while carbamazepine and metamizole metabolites were recurrently prevalent in water samples. Balakrishna et al. [63] reviewed up to 19 published research articles reporting on the occurrence of pharmaceuticals in Indian aquatic environments mainly activated sludge wastewater treatment plants (ASWWTPs), hospital WTPs, rivers and groundwater and found various PACs from a different group of chemicals detectable in Indian WTPs that mostly treat domestic sewage. Reif et al. [64] conducted a study on the occurrence of emerging micropollutants (EMPs) in groundwater and streamer sediment in Pennsylvania, about 7 PhCs including acetaminophen, caffeine, carbamazepine, tylosin, sulfamethoxine and sulfamethoxazole oxytetracycline were discovered in stream water samples collected from six paired streams sampling sites located upstream and downstream from animal feeding operations. The concentration of antibiotics ranged from 135 to 329 ng/L in the sample from the downstream site Snitz Creek in Lancaster Country. Vidal-Dorsch et al. [65] investigated the abundance of compounds of emerging concern (CECs) in Southern California coastal waters municipal effluents and marine water. Among several PhCs, naproxen, gemfibrozil and atenolol were the most frequently detected in concentrations above 1 µg/L. However, the impact of these contaminants on aquatic organisms and humans needs further investigation. In Africa, Anderson and Westrom [66] investigated the occurrence of pharmaceutical toxins in irrigation water from wastewater in Chirapatre Estates in Kumasi, Ghana. The results demonstrated that malaria treatment medication and paracetamol were detected in irrigation water derive from wastewater. Olarinmoye et al. [67] investigated the occurrence of antibiotics, oestrogens and lipid-lowering residues in surface water sewage from a wastewater treatment plant in Lagos, Nigeria; 37 pharmaceutical residues in the concentration range of 8.84 to 560 μg/L were observed. it was recommended that extended environmental monitoring of PhCs in Nigerian regions with high population densities need to be implemented. Matongo et al. [68] investigated the possible existence of pharmaceutical residues in water and sediments of the Msunduji River in Kwazulu-Natal, South Africa. The investigation showed that different groups of pharmaceuticals mainly antipyretics, antibiotics, antiepileptic and antipsychotic drugs were detected in the samples. From all groups, ibuprofen was the most abundant residue in wastewater (117 µg/L), surface water (84.60 µg/L) and sediments (659 ng/L). A similar study was carried out by Agunbiade and Moodley [69] that detected 8 acidic pharmaceuticals in wastewater, surface water and sediments from Msunduzi River, Kwazulu-Natal, South Africa. The study proved that aspirin and nalidixic acid were the most abundant drugs observed in the range of 118 µg/L and 25.2–29.9 µg/L, respectively. These studies demonstrate that pollution of water by pharmaceutical toxins is a global crisis that needs to be given attention even though the effect of these pollutants on humans is still under investigation, their presence in aquatic media may induce health issues. An extended global estimation of studies claiming the existence of PhCs in water sources is summarised in Table 1 and shows the estimated global abundance of pharmaceutical contaminants in water sources. Low occurrence of pharmaceuticals was recorded in Africa while the abundance of pharmaceutical pollutants in Europe, America and Asia reported in literature might be because environmental studies in those areas are more comprehensively done as compared to Africa; where there are insufficient funds and scarcity of sophisticated equipment to detect PhCs from water sources. Consequently, pharmaceutical substances have accumulated in water and wastewater matrices making their persistence a critical issue for the last two decades. Based on these reports, a huge number of treatment methods have been developed [52,70,71,72,73,74,75]. Most of these environmental remedies were found effective however, they offer high operational cost and lack of feasibility and their scalability constitute global constraints. In industries and water and wastewater treatment plants (WWTPs) the production of toxic sludge has been a shocking challenge that requires extended treatment methods before being discharged into the environment. Effluents from water were found to be contaminated with new emerging toxins such as pharmaceutical compounds and other related toxicants that are often present in extremely small concentrations [59,76,77,78]. This indicates that water from the tertiary stage needs to be treated before being distributed to different receivers/recipients. The persistence of these pollutants is certainly due to their accumulation in treatment facilities. It could also be inferred that the existence of these xenobiotics in minute concentrations in water sources has been proved long ago but their effect on humans and their ecosystems was neglected. As their effects on aquatic ecosystems are believed to be detrimental, this in return may also threaten human health even if their impacts on human genomes are still being studied.

### 2.1. Types of Pharmaceutical Remains in Water and Wastewater Sources

Antibiotics and their occurrence in the environment were reported in higher mg/L in hospital sewages, minor mg/L in municipal effluents, and ng/L in the surface, ground wastewater, and in tap water, respectively [102]. A particular group of pharmaceuticals has attracted environmental scientist’s attention due to their resistance to bacteria in WWTPs [100]. For example, antibiotics such as sulfonamides and fluoroquinolones may be toxic to aquatic creatures and their phytotoxic behaviour might be problematic to the environment. In addition to antibiotics, Non-Steroidal Anti-Inflammatory Drugs (NSAIDS) is another class of pharmaceuticals that exhibit pain-relieving, antipyretic and anti-inflammatory properties [103]. The presence of pharmaceuticals such as aspirin, acetaminophen, diclofenac, ibuprofen, naproxen, ketoprofen has been observed in drinking water. Clofibric acid commonly used as blood lipid regulator has also been currently reported. This active metabolite has been detected in wastewater treatment plants, groundwater, and tap water in tens of mg/L [78]. This is probably due to its persistent characteristic and high mobility in the aquatic environment [78]. Gemfibrozil and bezafibrate have also been identified in surface water. An additional group of antihypertensive drugs often used to lower blood pressure include beta-blockers such as metoprolol, atenolol propranolol, and diuretic furosemide, as well as ACE inhibitors, calcium channel blockers, and angiotensin II receptor antagonists, have repeatedly been detected in tens of mg/L in the ground, surface and drinking water [104,105,106]. An additional pharmaceutical active compound known as carbamazepine mainly used for epilepsy treatment has been detected in groundwater and drinking water sources in tens of mg/L. Carbamazepine metabolites are stubborn in WWTPs and these toxins often end up detectable in drinking water supplies [107] as well as primidone another antiepileptic detected in surface water and drinking water. Pharmaceuticals used in hormone-replacement therapy and oral contraceptives in veterinary medicine focusing on growth enhancement such as 17 β-estradiol, estrone and 17 α-ethinylestradiol were identified in the river, ground and drinking waters [108]. The impact of these endocrine disruptive compounds (EDCs) on aquatic organisms were also highlighted. The pharmaceutical compounds reviewed above considered as the principal types of water contaminants and were presented in Table 2. As a consequence of the growing accumulation of persistent organic pharmaceuticals, their removal from different water sources using advanced oxidation processes constitutes an essential component of research and development in water and wastewater treatment sectors [109,110,111]. Research studies on surface water treatment have shown that pharmaceuticals are non-biodegradable in sewage treatment plants [112]. The direct discharge of untreated municipality wastewater, manufacturing effluents, and indiscriminate dumping of unused medicines remain the principal outlet for pharmaceuticals prevalent in the environment as summarized in Table 2 [113]. Based on the effects of pharmaceuticals on humans and aquatic species, various water treatment methods such as filtration, coagulation/flocculation, and biological, and adsorption (activated carbon), ion exchange and reverse osmosis have been applied. However, these methods might require a lot of wet chemistry and the equipment required for their scale-up could be expensive and could limit their application in the remediation of pharmaceutical compounds. Therefore, extended efficient treatment procedures are required for water purification. Plasma technologies plus combined advanced oxidation techniques could promote direct mineralization of pharmaceutical pollutants in aquatic media.

### 2.2. Effect of Pharmaceutical Substances in Aquatic Environments on Health

The disproportionate utilization of numerous types of pharmaceutical substances has increased the direct or indirect contamination of water sources and the bio persistence of these pharmaceutical remains in water sources offer potential risks on the modification of living organism genomes and possibly long term effects on human health [59,114].

Urban wastewater polluted with medical substances has been identified as one of the major sources of pharmaceutical toxins [71] as well as the pharmaceutical contamination derived from pharmaceutical industries and the dumping of unused drug excesses in hospitals and households. Martín et al. [115] also reported that human excretions containing defecated pharmaceuticals circulate through the sewage system and end up in wastewater treatment plants (WWTPs). These were viewed as one of the principal sources of water pollution. Jones et al. [71] and Sirés and Brillas [114] reported that the recalcitrant behaviour of pharmaceuticals in water is because after biodegradation, deconjugation, sorption, and photodegradation processes, up to 90% of pharmaceutical residues consisting of unmetabolised and metabolized bio recalcitrant fragments were still present in final effluents of water and wastewater treatment plants (WWTPs). As WWTPs technologies have not been designed for such minute compounds, they are therefore transferred to receiving surface waters such as rivers and even to drinking water [55,78]. WWTP slurry containing nondegradable pharmaceuticals is also utilised as manure that contaminate surface water when emitted veterinary pharmaceuticals via fertilizer application is run-off and leaching takes place, affecting ground and surface water. Water contamination largely contributes to environmental pollution across the globe due to its significant impact.

Kümmerer [59] stated that the release of toxic water into the environment could potentially impact the biological systems and a few studies have highlighted irrevocable alterations caused by pharmaceutical contaminants in some micro-organisms [110,116]. The unexpected genomic changes in bacteria make them even more resistant towards pharmaceutical biotoxins as the unmetabolised and metabolized pharmaceutical remainders agglomerate and often form complexes such as beta-blockers whose toxicity has rarely been anticipated [117].

Pharmaceuticals can transform to endocrine (EDCs) which are well known to inhibit human genetic systems and as a result, the existence of carcinogenic pharmaceuticals in water treatment facilities may eventually obstruct the distribution of clean water to receivers. The complete decomposition of pharmaceuticals is mandatory to avoid potential health risk on human beings.

The appearance of pharmaceuticals in influents and treated effluents shows that traditional bioremediation and physicochemical remediation methods such as coagulation, volatilization, adsorption, sedimentation, and filtration are incompetent to fully destroy these emerging disruptors [71,72]. Even though chlorination and UV irradiation processes are often used for disinfection of final effluents in treatment plants [72,78], robust oxidative systems/protocols are crucially required for the total removal of pharmaceutical pollutants.

## 3. Overview of the Plasma Process

**Figure 1:**
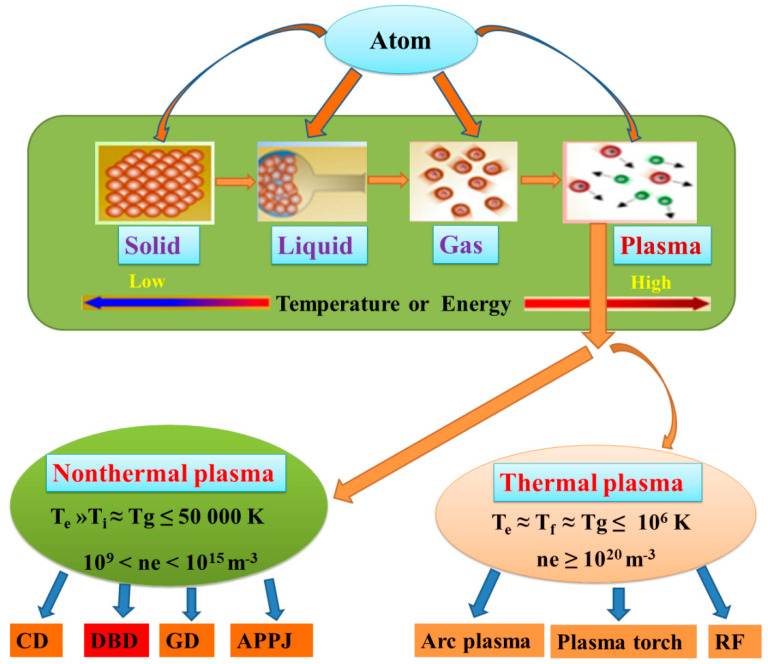
Plasma discharge and common configurations [119,120].

Solid, liquid, gas and plasma have been widely recognised as the principal states of matter for decades. Currently, plasma often known as the fourth state of matter is derived from gas through ionisation, dissociation, and excitation of atoms and molecules rigidly bonded in the gaseous state. According to Akishev et al. [118] plasma consists of various gaseous particles such as energetic electrons, ions, and neutral active species mainly gaseous molecules, free radicals, metastable species and UV-photon. Plasma can be categorised into thermal plasma and non-thermal plasma as shown in Figure 1.

### 3.1. Thermal Plasma

Thermal plasma (TP) is an electrical discharge process that occurs at high temperatures (T ≈ 300–10^6^ K ≈ 26.85–49,726.85 °C). In thermal plasma, the electron temperature is approximately equal to that of ions and gas temperature (T_e_ ≈ T_i_ ≈ T_g_ ≈ 15,000–10^6^ K ≈ 14,726.85–999,726.85 °C, corresponding to the ionization energy range of 10–15 eV). One of the major advantages of TP is the production of highly energised electrons with an approximate density ne ≥ 10^20^ cm^−3^ [121]. Thermal plasma is often induced by various electrical discharges including free burning arcs, transferred arcs or non-transferred plasma torches, AC or transient arcs (lamps, circuit-breakers, or pulsed arcs); radiofrequency (RF) and microwave (MW) discharges at near-atmospheric pressure; and laser-induced plasmas (LIP) [122,123,124,125]. Hur et al. [126] investigated the characteristics of TP by numerical and experimental modelling of transferred plasma torches at atmospheric conditions to determine the effect of working parameters and their corresponding electrode arrangements on waste remediation. The results showed that for any TP torch configuration, optimisation of operating variables is necessary to achieve desirable waste conversion and following Yuan et al. [127]. Thermal plasma configurations have extensively been used in diverse aspects of environmental remediation. Sobacchi et al. [128] investigated the production of hydrogen via partial oxidation/reforming of liquid gasoline-like fuels by thermal plasma/catalytic system. The authors claimed that the catalyst demonstrated excellent activity in terms of hydrogen production at temperatures greater than or equal to 800 °C (1073.15 K). On the other hand, the oily waste sludge (OWS) is a toxic residue regularly produced in petrochemical industries and is considered as a harmful waste that requires absolute treatment. Torres et al. [129] applied a non-transferred plasma torch (NTPT) and transferred plasma torch (TPT) for the treatment of OWS at the applied conditions (OWS solution injection feed rates varied from 0.25 to 5.8 g min^−1^, plasma torch power ranging from 1 to 10 kW and plasma gas flow rate of 20 m^3^ h^−^^1^). Their outcome indicated that 99.99% degradation of organic substances and 95.5% volume reduction were achieved. The gaseous residual by-products obtained had low molecular weight and were claimed to be non-hazardous and could be discharged in landfills. This was further complemented by Fabry et al. [130] who reported that thermal plasma induced by DC or AC plasma torches is an adequate method for the conversion of waste to energy and can certainly be used for the remediation of numerous wastes including municipal solid wastes, heavy oil, used car tires and medical wastes by gasification processes. On the other hand, Chandran et al. [131] investigated the efficiency of radio frequency (RF) plasma on nitridation that involved the incorporation of nitrogen onto the surface of the polycrystalline diamond at varied temperatures (250, 500, 750, 1000 °C). The results of their research showed that nitrogen content in the diamond surface declined with an increase in temperature during the nitridation process. Nevertheless, the incorporation of nitrogen into the diamond surface by RF plasma was successful. Recently, Belov et al. [132] studied the dissociation of carbon dioxide in a microwave plasma reactor operating in a pressure range of 200 mbar–1 bar and gas inlet configurations. Their results showed that in the direct flow configuration, the lowest conversion ~3.5% at 200 mbar and 2% at 1 bar were obtained at exhaust temperatures of up to 1000 K (726.85 °C). Alternatively, the highest conversion of 38% at 200 mbar and 6.2% at 1 bar, with energy efficiencies of 23% and 3.7%, respectively were achieved during the post-discharge cooling step introduced for the reverse gas inlet regime. The studies reviewed above showed that thermal plasma and its various properties and configurations are effective for solid and liquid wastes recovery, hydrogen production, CO_2_ conversion, etc. that perhaps other treatment systems would not be able to achieve. However, it could be noticed that most of these investigations were carried out in the temperature range of 250 to 1000 °C (523.15–1275.15 K) or even higher than these values and hence are energy demanding. Also, the scalability of TP configurations to the industrial level to treat large volumes of water and wastewater could be costly. So, handling such high-temperature values at small lab scales for water and wastewater treatment could be challenging and contradictory to the development of ideal low energy consumption technologies.

### 3.2. Non-Thermal Plasma

Non-thermal plasma (NTP) refers to electrical discharge occurring in the temperature range of 300–50,000 K ≈ 26.85–726.85 °C). In non-thermal plasma, the electron temperature is much greater than the ions and gas temperature (Te » Ti ≈ Tg ≈ 10,000–50,000 K ≈ 14,726.85–49,726.85 °C, with a corresponding ionisation energy range of 1–10 eV and electron density in the range of 10^9^–10^15^ cm^−3^). Non-thermal plasma is subdivided into four categories namely corona discharge (CD), dielectric barrier discharge (DBD), glow discharge (GD), and atmospheric pressure plasma jet discharge (APPJ). The plasma properties presented in Figure 1 are based on the temperature difference between thermal (≤10^6^ K) and non-thermal (≤50,000 K) parameters. Although various AOPs have been widely used for the effective removal of water contaminants/organic pollutants, the incomplete degradation of pollutants leading to toxic intermediates can result in detrimental effects than parent compounds; hence alternatives for total oxidation of targeted contaminants are highly required.

Non-thermal plasma (NTP) in its diverse configurations has been widely used in various sectors for waste treatment and decontamination purposes. Murugesan et al. [133] reviewed the physical and chemical properties of non-thermal plasma and their application in the removal of organic pollutants and microbes. The authors conveyed that the chemical cocktail consisting of UV radiation and diverse reactive species render non-thermal plasma an adequate treatment method that is economically feasible and has been applied in various areas of environmental remediation. NTP used in combination with adsorbents, catalysts, electrolysis, biodegradation, ultrasonication, ozonisation and ultrafiltration processes could be promising methods for the removal of persistent pollutants and microorganisms from polluted waters, and this was also endorsed by Zeghioud et al. [134].

For instance, Zeng et al. [135] studied the degradation of ibuprofen (IBP) pharmaceutical drug in aqueous solution by cylindrical wetted-wall corona discharge reactor. In their study, the effect of parameters including initial concentration, pulse repetition rate were investigated. The outcome of their study showed that the highest degradation of 91.7% of 60 mg/L IBP was achieved after 80 min of plasma exposure corresponding to an energy yield of 6.9 g/kW h. IBP degradation followed the first-order reaction rate of 30.3 × 10^−^^3^ min^−^^1^. The resultants IBP by-products identified by high liquid performance chromatography (HPLC) were amenable and their degradation mechanistic pathways show that IBP was almost totally decomposed to H_2_O, CO_2,_ and simpler salts. Another investigation focusing on oxidation of a wide range of pharmaceuticals from real wastewaters (raw sewage from a public hospital and biologically treated wastewater of a health-care institute) by gas-phase pulsed corona discharge was carried out by Ajo et al. [136]. Their results showed 87% removal of pharmaceutical residues from raw sewage with reasonable energy consumption of 1 kWh^−3^ while 99.99% removal of pharmaceutical toxins in biologically treated wastewater was reached at low energy of 0.5 kWh^−3^. A similar study involving the treatment of wastewater containing various pharmaceuticals including carbamazepine, diatrizoate, diazepam, diclofenac, ibuprofen, 17α -ethinylestradiol and trimethoprim by corona discharge was also conducted by Banaschik et al. [137] and enhanced the removal percentages of pharmaceuticals up to 99.99%. Extended use of corona discharge for the removal of pharmaceuticals in wastewater effluents has been outlined in the literature [138,139]. However, it can be pointed out that in these corona discharge configurations the high voltage electrode (HV) is likely in direct contact with the effluent being remediated and could be attacked by oxidative species produced in solution.

On the other hand, Gadri et al. [140] developed atmospheric uniform glow discharge plasma that was tested for sterilisation and surface decontamination. The authors informed that this discharge is typical non-thermal plasma with conventional features like DC normal glow discharge that operates in air at low pressure. Gadri et al. [140] claimed that their plasma system generates various active species responsible for surface disinfection and sanitization. They reported that their technology is feasible, cost-effective, and appropriate for the cleaning of three-dimensional workpieces such as medical instruments. Glow discharge plasma has been used for various purposes from food packaging and screening of contaminants in foodstuff to fabrication, modification and treatment of nanomaterials with improved properties [141,142,143,144,145].

Conversely, Rashid et al. [146] investigated the treatment of textile wastewater by underwater parallel-multi-tube air discharge plasma jet with an input power of 16 W, voltage varied from 0 to 10 kV at a frequency of 4 kHz. The model wastewater used in their study consisted of three types of industrial-grade textile dyes including remazol blue (RB), remazol red (RR) and remazol yellow (RY). The results showed that almost 70% degradation of all dyes was achieved within 10 min of plasma treatment while the remaining 30% required prolonged plasma exposure to reach completion. The authors found that during the treatment process, solution pH decreased, while the conductivity increased with the increase of treatment time. The three reactive species O_3_, H_2_O_2_ and NO_3_^−^ were identified by optical emission spectroscopic (OES) and their concentration decreased in the plasma system during treatment, hence showing their involvement in the degradation of the model dye pollutants. The authors further show that amines and alkynes were likely the main by-products that were formed via oxidation of azo-bonds and nitrogen-containing functional groups probably induced by O_3_ and OH^.^ [54]. The longer time required to reach total degradation of dyes beyond 10 min of plasma treatment was certainly due to the recalcitrance behaviour of the degradation intermediate by-products in the effluent that was being treated. Thereafter, the authors claimed that their technology is environmentally benign and could be explored in wastewater treatment facilities. Similar investigations using plasma jet for wastewater remediation have also been reported in the literature Xin et al. [147]. Apart from this, plasma jet has also been used in mass spectrometry analysis for direct detection of compounds from surfaces and complex matrices [148].

Marković et al. [149] studied the degradation of ibuprofen (IBP) by falling water film dielectric barrier discharge reactor set at the applied voltage of 17 kV, frequency of 300 Hz, and a 65 W with and without homogenous catalyst (Fe^2+^). The experiments were carried out at atmospheric pressure and room temperature. The results of their study showed that, after 15 min of plasma run, 80% degradation of 60 mg/L IBP was achieved with DBD alone while 99% decomposition of IBP at the same initial concentration was reached with DBD combined with Fe^2+^ catalyst. The liquid chromatography-mass spectrometry time of flight (LC-MS TOF) analysis of the treated water showed that nine degradation intermediate by-products were obtained with DBD alone and four with DBD/Fe^2+^. The toxicity effect of the treated effluent determined with two bioassays: Vibrio fishery and Artemia salina showed that the resultant by-products were amenable. A comparable study was conducted by Tang et al. [150] on the degradation of oxytetracycline (OTC) antibiotics using a gas phase dielectric barrier discharge (GPDBD) plasma reactor at the following conditions: applied voltage 7.5 kV, airflow rate 1.0 L/min, initial OTC concentration 100 mg/L, and initial pH 5.0. The outcome showed that after 20 min of plasma discharge experiment, 93.4% of OTC degradation was achieved at the applied conditions. The decomposition of OTC in the DBD system with time was followed by UV-vis and HPLC-MS. The TOC and COD removal efficiencies 43.0% and 73.7%, respectively demonstrated that the resultant degradation metabolites of OTC were less harmful, and the proposed elimination mechanism pathways demonstrated that OTC was degraded to H_2_O, CO_2,_ and simpler entities. Complementary investigations on the removal of pharmaceutical residues in water by DBD technologies have been reported [134,151,152].

The application of non-thermal plasma for water and wastewater remediation showed that most studies reviewed above were conducted at atmospheric pressure and room temperature with less power consumption and hence reduced energy demand. This is manageable as compared to thermal plasma whose operational atmospheric conditions often involve high-temperature fluctuation between 300 and 50,000 K that might be energy demanding and challenging to handle at small scale water and wastewater treatment laboratories. In comparison, it could be inferred that TP is efficient in recycling solid and liquid wastes and vital for research and development focusing on understanding its properties and limitations. In contrast, for immediate urgencies in water and wastewater treatment facilities, NTP technologies requiring less energy and operating at normal conditions of temperature and pressure could be suitable alternatives for water and wastewater purification.

Still, NTP induced by various electrical discharges encounters some limitations. For example, although the corona discharge electrode arrangement has been shown effective for the remediation of contaminated water, direct exposure of the high voltage (HV) electrode to the solution being treated often results in its corrosion and hence the discontinuation of the treatment process and extended contamination of the treated effluent. Conversely, glow discharge and plasma jet have demonstrated excellent capacity for surface cleansing, water, and wastewater purification to some extent. However, the scalability of these electrode geometries to large treatment units (semi or industrial levels) for the treatment of thousands of litres of polluted water could be costly and problematic.

Alternatively, in DBD technologies the HV electrode is protected by one or more dielectric barriers that ensure long-term production and circulation of highly energized electrons whose contact with feed gas generates diverse reactive species that effectively combat and convert the targeted pollutants directly into H_2_O, CO_2,_ and related harmless by-products. Extended prominence of DBDs over other configurations relies mostly on the abundance of highly energized electrons freely circulating the dielectric barrier tube at minimal energy of 1 eV. Its feasibility at room temperature, scalability, and flexibility renders DBD one of the most potentially applicable treatment techniques for water and wastewater purification. These advantages of DBDs have therefore attracted worldwide attention for their use in water and wastewater treatment. Since the accumulation of pharmaceutical residues in polluted water has continuously increased in the past decades as a result of excessive consumption of medications coupled with the up-surging world population, the removal of pharmaceutical toxins in polluted water is of urgent interest. Therefore, the application of DBDs for complete decomposition of pharmaceutical contaminants in water effluents has gained global attention. Hence this review discusses various studies dedicated to the removal of pharmaceuticals by DBD technologies.

#### 3.2.1. Dielectric Barrier Discharge: General Description

Non-thermal plasma based on DBDs usually referred to as silent discharges have emerged as novel remediation technologies and have been employed for various applications including ozone generation, control of gaseous toxins, and the demolition of carcinogenic compounds [153,154]. DBD configurations aim at the generation of various molecular and radical species such as O_3_, H_2_O_2_, O**^.^**, O_2_**^.−^**, OH, electrons and UV photons that directly or indirectly decompose hazardous compounds. The priority of DBDs is the production of non-selective hydroxyl radicals that is an exceptionally effective oxidant, with an oxidizing potential of 2.8 V higher than that of ozone 2.07 V [153]. The OH radical is the most dominant oxidative species that non-selectively attacks recalcitrant contaminants and converts them into dissolved CO_2_, H_2_O, and other harmless substances.

The most common DBD electrode configurations involve either single or double planar and cylindrical dielectric-barrier discharges [153,154]. These configurations consist of one or more insulating layers often referred to as a dielectric barrier between metal electrodes and discharge gap(s). The presence of more dielectric boundaries in DBD configurations not only minimizes the risk of electrode corrosion but intensifies the existence of electric and magnetic fields to ensure the even distribution of highly energized electrons around the dielectric materials even though the upscaling of these configurations has always been challenging.

#### 3.2.2. Summary of the Dielectric Barrier Discharge Operational Scheme

DBD involving the generation of non-selective hydroxyl radicals is a promising tool for effective degradation and mineralization of target pollutants in water and wastewater [20,43]. Apart from the production of OH radicals, the DBD system also generates UV light, O_3_ and H_2_O_2_ and their combination in aqueous media allow water and wastewater treatment without the addition of chemicals. Even though various DBD configurations have been employed in the decomposition of pharmaceuticals in aquatic media [155,156,157,158,159,160], their efficiency may depend on their electrodes geometries. A typical DBD reactor described by Mouele et al. [54] consists of the inner and outer quartz dielectric barriers/tubes and the region between the two tubes is often referred to as the air gap or discharge zone. The two tubes are sealed at the top of the outer part of the reactor. At the bottom, the second barrier is sealed to a porous sparging outlet. The inner tube diameter is often around 1 mm and that of the outer tube is about 7 mm. The DBD reactor (e.g., 23–25 cm) long with an inlet and outlet for air circulation and an air gap of about 2 mm. The simulated wastewater placed in the reactor is considered as the ground electrode and earthed to complete the circuit. The air compressor with a controllable flow rate (using an airflow meter) is connected to the gas inlet. The feed gas fed from the inlet freely circulates through the plasma zone and is uniformly spread as bubbles into the simulated wastewater via the porous sparging material to achieve maximum oxidation of the pollutant. The set voltage, airflow, pollutant volume, and other parameters are either varied or kept constant for optimisation purposes. A power supply set at a voltage delivering a certain amount of current is directly connected to a high voltage electrode that is immersed in an electrolyte solution placed in the inner tube of the reactor. The interaction between the highly energised electrons around the inner tube and air gas (N_2_ and O_2_) or pure oxygen (O_2_) induced the formation of UV light and various exciting unstable species such as N*, O**^.^**, O_3_, etc. in the plasma zone. The generated species in the air gap follow an avalanche of chemical reactions and are directly circulated into the polluted water, where secondary species such as H_2_O_2_ and OH^.^ are subsequently produced.

#### 3.2.3. Degradation of Pharmaceuticals by DBD Configurations

Dielectric barrier discharge (DBD) technologies have been extensively used for the decontamination of pharmaceutical contaminants from water and wastewater. For instance, Kim et al. utilised DBD to decompose veterinary antibiotics such as lincomycin, ciprofloxacin, enrofloxacin, chlortetracycline, oxytetracycline, sulfathiazole, sulfamethoxazole, sulfamethazine, and trimethoprim in aqueous solutions. The influence of various parameters such as the type of gas used (air, O_2_) and gas flow rates, initial concentration of contaminants and the discharge power on the decomposition of the targeted antibiotics was assessed. The results showed that antibiotics decomposed differently, and their degradability depended on the amount of energy provided and hence followed an exponential decay for the energy delivered in the DBD system. The effect of the evaluation of the parameters showed that at an optimum concentration of 5 mg/L, the energy required ranged is between 0.26 and 1.49 kJ/mg that corresponded to 60% of degradation. Likewise, about 0.39 to 2.06 kJ of energy was required to achieve 90% degradation at the same concentration. Table 3 presents a summary of the degradation of pharmaceuticals by DBD systems. It is worth noting that the diagnostic of configurations used to achieve significant removal percentages was not clarified [113].

**Figure 2:**
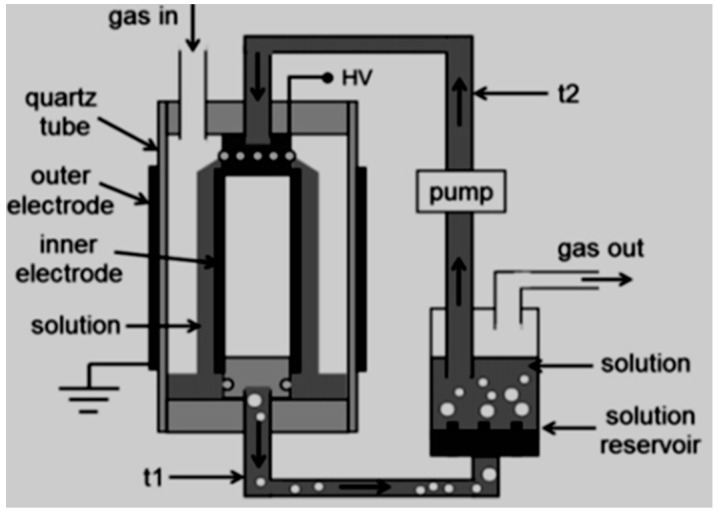
Single dielectric barrier discharge (DBD) reactor used for the decomposition of pentoxifylline and antibiotics in water [155].

Subsequent research investigation by Magureanu et al. [113] on the degradation of pharmaceutical compound pentoxifylline in water using a continuous semi-batch single dielectric barrier discharge reactor (Figure 2) at normal conditions of temperature and pressure revealed that after 60 min, about 92.5% of pentoxifylline removal was achievable.

A similar DBD configuration was employed for the oxidation of three β-lactam antibiotics (amoxicillin, oxacillin, and ampicillin) in water. The outcome showed that after 30 min, about 90% of amoxicillin and oxacillin were oxidised and after 120 min more than 90% of ampicillin degradation was reached.

**Figure 3:**
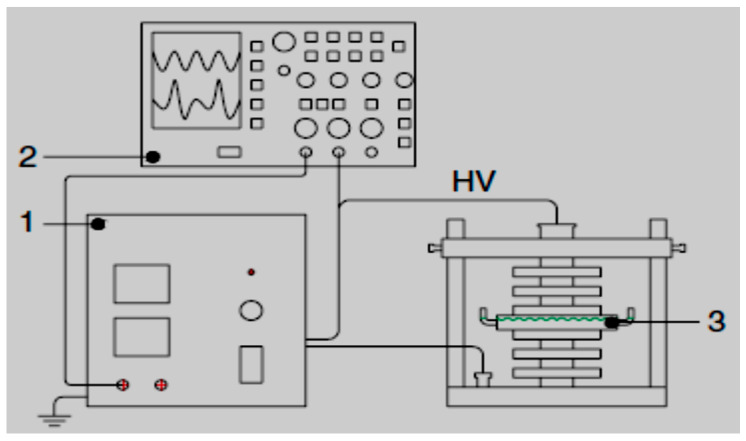
Experimental setup of the dielectric barrier discharge (DBD) water treatment process: 1. High voltage (HV) generator, 2. Oscilloscope, 3. Quartz reactor [156].

Some pharmaceuticals have been proved non-degradable by AOPs as well as in conventional treatment methods. A typical example focuses on both ionic and nonionic ICM-iopromide claimed to be recalcitrant to ozone and traditional wastewater treatment plants [156]. Liu et al. [156] investigated the removal of a unique kind of ICM-iopromide (IOPr) using a dielectric barrier discharge (DBD) reactor (Figure 3), no decrease in total organic carbon (TOC) was observed. Likewise, using FT-IR spectra proved that the degradation of the IOPr molecule was mainly induced by hydroxylation, carbonylation, and deiodination processes. The increase in the BOD5/COD ratio indicated that the IOPr compound is biodegradable and its biodegradability follows first-order kinetics while the resulting by-products of IOPr after DBD treatment were easily absorbed and biodegraded by activated sludge. DBD was thus proven to increase the biodegradability of recalcitrant pollutants.

It is however crucial to clarify the difference between the terms degradation and mineralization. During the degradation process, the targeted contaminant is subjected to selective oxidative species such as ozone, or to a non-selective radical like OH**^.^** which may undergo reaction mechanisms such as ring-opening. This in turn might lead to the formation of either bigger molecules than the parent compound or smaller entities. The small by-products produced in the DBD system can then be further oxidised to dissolve CO_2_, water, and simpler salts, often indicated by a decrease in TOC content, and such process is often referred to as mineralisation which is the principal goal of most advanced oxidation processes.

Alternatively, the bigger or smaller by-products can react to yield new substrates with even higher molecular mass and often referred to as oligomerisation. In other words, in the DBD configuration used by Liu et al. [156], IOPr was degradable as shown by the 98.8% decomposition percentage obtained and biodegradable as indicated by the increase of BOD5/COD ratio. Likewise, the recalcitrant change in TOC content demonstrated that IOPr was not mineralised. Therefore, an improvement of the DBD system employed in their investigation might be necessary to yield more OH**^.^**, that represents the driving force of the mineralisation process. This confirms once again that an efficient DBD configuration should be capable of producing a huge amount of OH**^.^** in such a way that even if the targeted compound is selective towards certain oxidants, in the end, its mineralisation should occur [157,158,159].

**Figure 4:**
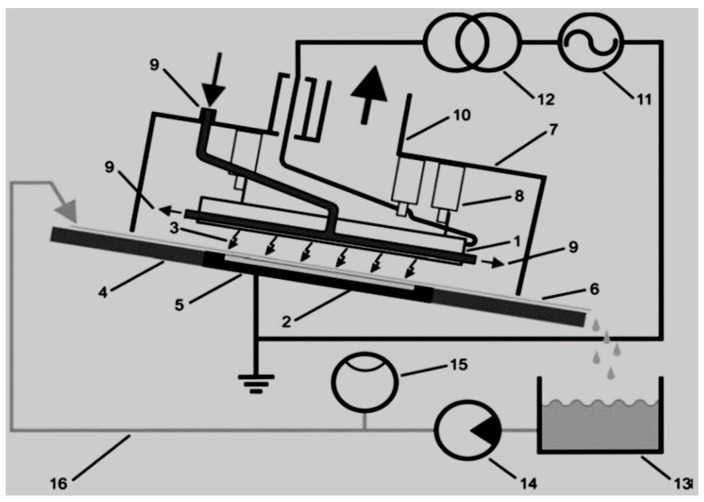
Diagram of the flow-through reactor. 1—barrier electrodes; 2—catalytic counter-electrodes; 3—corona discharge; 4—stainless steel plane; 5—PTFE isolator; 6—thin water film; 7—airproof case; 8—ceramic isolator; 9—gas inlet and the two injection points; 10—exhaust; 11—frequency generator; 12—high voltage transformer; 13—sample reservoir; 14—external gear pump; 15—flow control; 16—Teflon tube [161].

**Figure 5:**
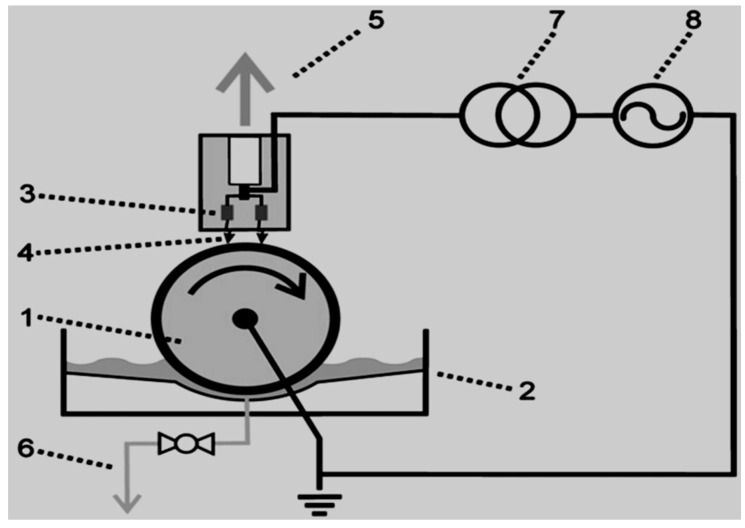
Diagram of the rotating drum reactor. 1—rotating drum; 2—sample reservoir; 3—barrier electrodes; 4—corona discharge; 5—exhaust; 6—sample drain hose; 7—high voltage transformer; 8—frequency generator [161].

Krause al. [160] studied the degradation of persistent pharmaceuticals in aqueous solutions by a positive dielectric barrier discharge treatment exhibited in Figure 4. Results showed that the model pollutants carbamazepine, clofibric acid, and iopromide could be decomposed as a single solution mixed with ultra-pure water. Clofibric acid and iopromide were decomposed after 30 min, while 98% and 99% removal of carbamazepine and iopromide were attained, respectively. DBD configurations (Figure 5), modified by Krause et al. [161] was redesigned for the removal of pharmaceuticals from water and wastewater and to examine the degradation of endocrine-disrupting chemicals (EDCs) carbamazepine, clofibric acid, and iopromide by DBD-corona rotating drum discharge reactor over water. Previously, the three pharmaceuticals were selected at the same concentration 0.1 mM with different powers values ranging from 250, 500 to 750 W. In the first set of experiments, the three compounds were degraded individually. Results showed that after 30 min of the experiment, the degradation efficiency of carbamazepine and iopromide reached 94% and 98%, respectively at 500 W, while clofibric acid showed the highest removal.

In contrast, exposure to the mixture of the three compounds showed that the oxidative degradation of carbamazepine and clofibric acid reached 99% at 500 W after 90 min of treatment. Likewise, 67–75% of iopromide removal was achieved at the same conditions. These results obtained confirm the effectiveness of DBD configurations during the water treatment process. The difference in degradation percentages recorded when the three pollutants were degraded individually or in the mixture demonstrates that even though these compounds belong to the same class (pharmaceuticals), they have different physical and chemical properties. That is, some may be highly reactive with certain oxidants produced in the DBD system while others might resist the effect of oxidizing species and hence exhibit lower degradability. Therefore, to overcome this limitation, a performant DBD configuration could be designed. This in return may facilitate the generation of a significant amount of non-selective OH radicals that may directly convert contaminants into CO_2_, water, and inorganic salts.

**Figure 6:**
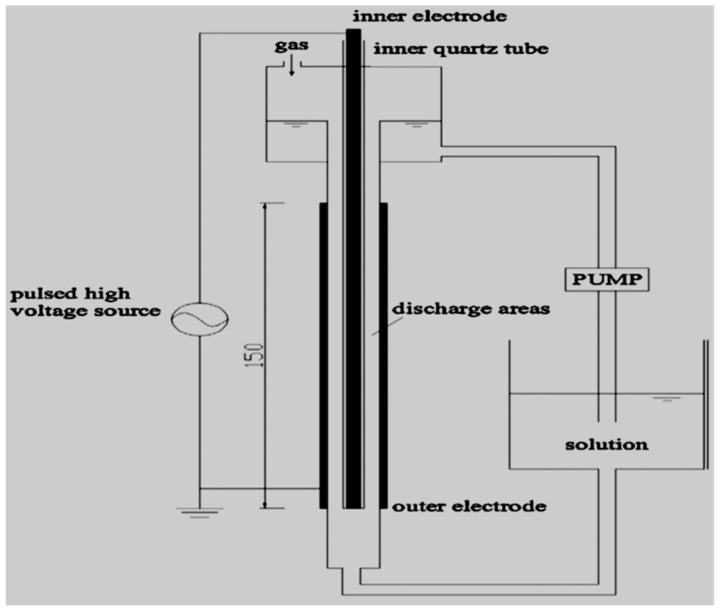
Single-cylinder dielectric barrier discharge reactor used for the degradation of sulfadiazine antibiotics [162].

**Figure 7:**
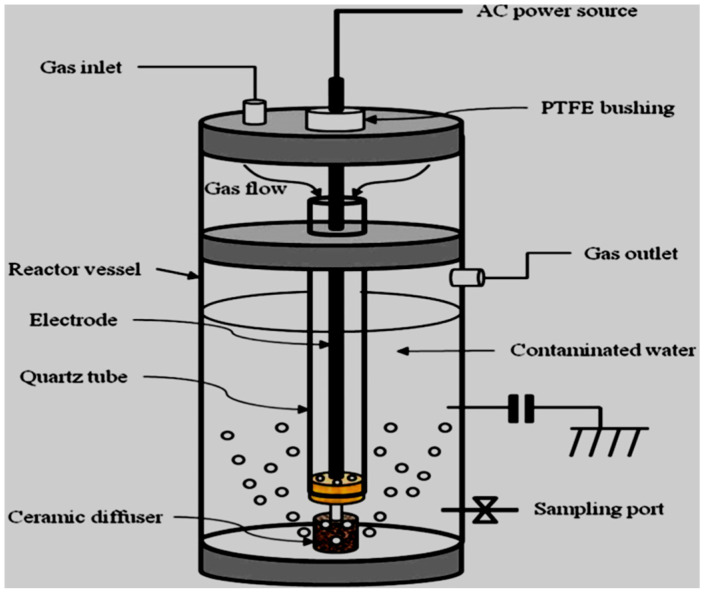
Experimental set up for the degradation of antibiotics [162].

Rong et al. [157] studied the degradation of sulfadiazine (SDZ) antibiotics by water falling film dielectric barrier discharge shown in Figure 6. During their experiments, the authors noted that the degradation of SDZ depended on output power intensity. Consequently, the decomposition of SDZ was evaluated at three different output intensities 100 W, 150 W and 120 W. Results showed that the highest degradation of SDZ was 96% achieved at 150 after 15 min of the experiment. Likewise, 87% and 94% of ZDS removal were achieved at 100 W and 120 W, respectively. Investigative studies of the impact of some parameters (initial concentration, gas type (air, O_2_) and gas flow rate) on the degradation of synthetic antibiotics mainly lincomycin, ciprofloxacin, enrofloxacin, chlortetracycline, oxytetracycline, sulfathiazole, sulfamethoxazole, sulfamethazine, and trimethoprim using dielectric barrier discharge (DBD) plasma system (Figure 7) showed that antibiotics were efficiently degraded and their removal was energy-dependent [160]. These results proved that the DBD configuration had potential as an efficient technique for water decontamination [162].

To recall, the occurrence of drugs in water sources can result in more toxic by-products that require urgent dismissal. This entails that the chemicals in pharmaceuticals and personal care products speedily transform into different complexes when they enter the environment and their toxic effects are moderately known. Extended studies have been devoted in the past half-decade for complete decomposition/oxidation of pharmaceutical toxins and metabolites.

**Figure 8:**
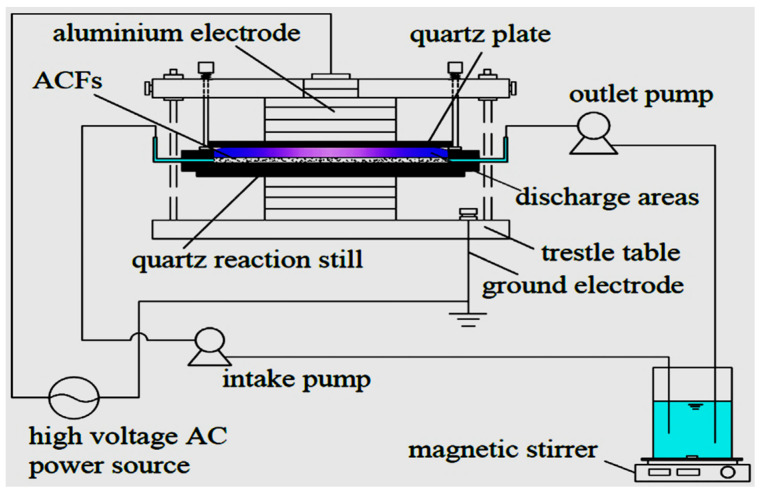
Reactor experimental set up utilised for the degradation of triclosan (TCS) [163].

For instance, Xina et al. [163] assessed the degradation of triclosan (TCS) by a single planar dielectric barrier discharge (DBD) plasma reactor presented in Figure 8. During their investigation, the effect of input power varied from 60, 80 to 100 W, TCS initial concentration changed from 5, 8 to 10 mg/L and airflow rate altered from 30, 45 to 60 mL/min upon the removal efficiency of TCS was examined.

The results of their research indicated that at the following fixed conditions: TCS with an initial concentration of 10 mg/L, airflow rate 45 mL/min and discharge time of 18 min, the degradation efficiency of TCS increased with the increase of input power and the highest of TCS removal 86% was achieved with 100 W at the applied conditions. On the other hand, at initial TCS concentration of 10 mg/L and input power of 80 W and discharge time of 18 min, the removal percentage of TCS rose with the increase of airflow rate, nevertheless the greatest TCS removal efficiency of 85% was attained with the airflow rate of 45 mL/min at indicated conditions. Moreover, at the input power of 80 W and an airflow rate of 45 mL/min and discharge time of 18 min, the impact of TCS initial concentration did not define any continuous trend, likewise, the highest TCS degradation efficiency of about 85% was reached with 8 mg/L TCS initial concentration at the applied conditions. Besides, Xina et al. [163] successfully proved that at the following optimum conditions, 10 mg/L TCS initial concentration, the input power of 80 W, airflow rate of 45 mL/min, and discharge time of 18 min, 85% of TCS removal was achieved with DBD alone. The total organic carbon (TOC) removal rate reached 12% at pH 6.26 indicating that the various TCS degradation intermediate by-products of TCS were thoroughly mineralised to dissolve CO_2_, H_2_O, and aqueous innocuous entities. Their outcomes clearly show that the DBD system alone is effective for the decontamination of pharmaceutical residues in water and wastewater.

**Figure 9:**
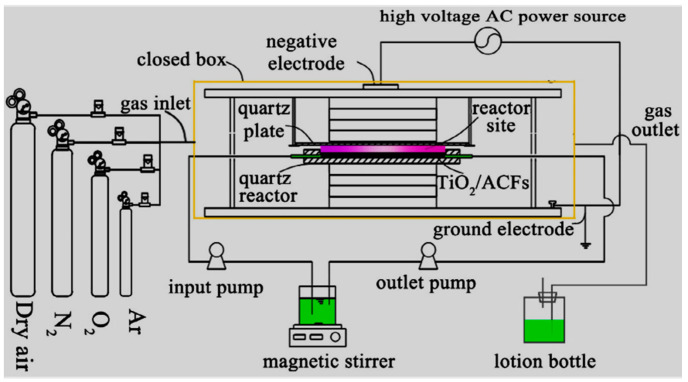
Experimental diagram of the dielectric barrier discharge (DBD) reactor used for the decomposition of triclocarban (TCC) [164].

A parallel study focusing on the decomposition of aqueous triclocarban (TCC) by dielectric barrier discharge (DBD) as disclosed in Figure 9 was conducted by Wang et al. [164]. During their investigation, the effect of initial concentration and output power upon the degradation of TCC was investigated. The outcomes of their study showed that at 10 mg/L TCC and discharge power of 38 W under air plasma exposure, the degradation rate of TCC reached 0.20 mg/L min^−^^1^ after 30 min with DBD alone. The authors further showed that at the experimental conditions, the mineralization efficacy of TCC reached 12% with the DBD system alone. Consequently, 64% of TCC toxicity removal was achieved with DBD alone after 30 min of plasma treatment. The GC-MS results showed that the refractory fragmentation by-products of TCC were converted to dissolved CO_2_, H_2_O, and simpler end products. Therefore, the study by Wang et al. [164] demonstrated that the DBD protocol is an excellent treatment technology even though the addition of catalyst at optimised conditions could improve the mineralisation of the targeted pharmaceutical pollutant.

**Figure 10:**
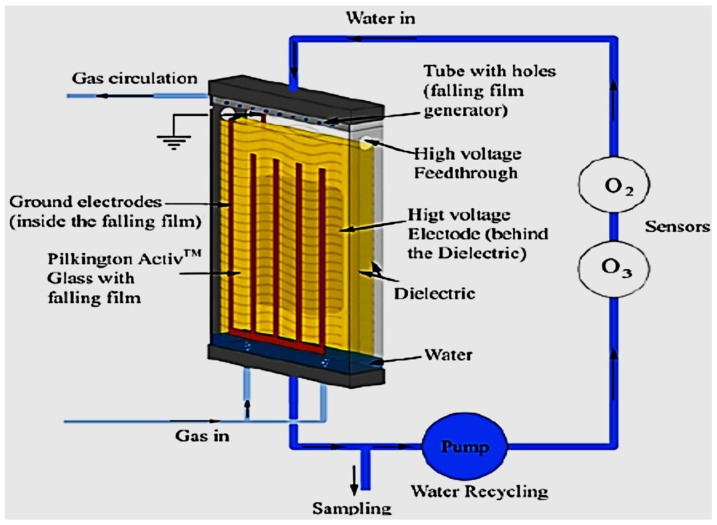
Planar water falling dielectric barrier discharge (DBD) reactor used for the degradation of diclofenac (DCF) and ibuprofen (IBP) pharmaceuticals [165].

Aziz et al. [165] used a planar falling film DBD reactor disclosed in Figure 10 for the degradation of the non-steroidal anti-inflammatory drugs (NSAIDs) diclofenac (DCF) and ibuprofen (IBP) in aqueous solution in comparison with other advanced oxidation processes including photocatalytic ozonation and photocatalytic oxidation. The results showed that at the following conditions: DCF and IBP initial concentration 50 mg/L each, discharge power 200 W under Ar gas atmosphere, complete degradation of DCF with DBD alone was reached after 20 min while that of IBP was achieved after 15 min of exposure to plasmas. The authors further showed that the combination of the DBD system with Fenton reagent in a gas mixture Ar/O_2_ enhanced the removal of the two drugs with total DCF decomposition attained after 10 min and that of IBP after 20 min of plasma treatment. These outcomes showed that both pollutants were differently decomposed under both DBD alone and DBD combined with photocatalyst systems. This could be attributed to the recalcitrance properties of DCF and IBP degradation intermediate by-products that certainly retarded their total mineralisation. The comparison of the DBD system with other AOPs showed that the abatement of DCF and IBP pharmaceuticals was adequate with photocatalytic oxidation and operative with direct ozonation in darkness. Moreover, the combination of ozonation and photocatalysis resulted in synergetic improved degradation and mineralization of both pharmaceuticals. Likewise, Aziz et al. [165] highlighted that the efficiency of their DBD system could be as effective as the AOPs but may largely depend on the feed gas used in the discharge process.

**Figure 11:**
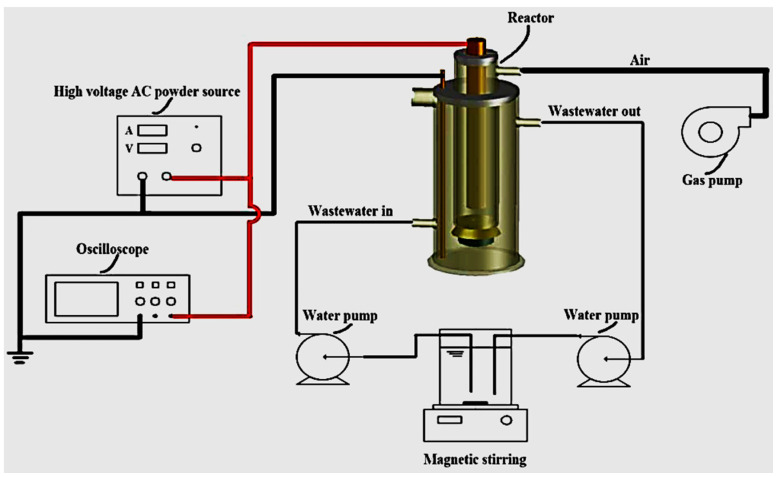
Plasma reactor set up used for the decomposition of acetaminophen (APAP) model pollutant [166].

Zhang et al. [166] tested the efficiency of a single axial dielectric barrier discharge system shown in Figure 11 upon the putrefaction of simulated acetaminophen (APAP) model water pollutants. During their investigation, the effect of the following parameters applied voltage varied from 18, 20, and 22 to 24 kV and solution pH changed from 4, 6.5, 7.5, 9 to 11.1 upon the removal of APAP was surveyed, distinctively. The outcomes of their study showed that at the applied voltage of 18 kV, 92% of 20 mg/L APAP degradation at pH 7.5 was achieved after 18 min of plasma exposure with DBD alone. The abetment of APAP resulted in three greener alcoholic and carboxylic acids by-products as revealed by UV–vis spectrometry and HPLC/MS analysis. These excellent results demonstrate the effectiveness of DBD technology in the remediation of pharmaceutical polluted water. However, authors should ensure that the anode electrode in the reactor geometry should be well protected and not corrode quickly to ensure the long-running capability of the plasma reactor.

**Figure 12:**
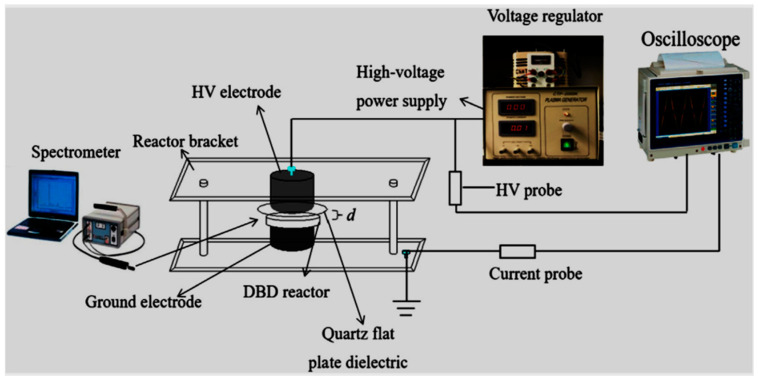
Dielectric barrier discharge (DBD) experimental system used for the decomposition of norfloxacin [167].

An accolade study on the degradation of norfloxacin (NOR) pharmaceutical was conducted by Xu et al. [167] using an air gas single planar DBD reactor disclosed in Figure 12. In their investigation, the authors focused on the determination of the aqueous concentration of major long-lived reactive oxygen species (ROS) including O_3_, H_2_O_2,_ and NO_3_^−^ when altering the discharge time varied from 0.5, 1, 2 to 4 min and initial solution pH changed from 3, 5, 7 to 9 in the DBD system. The results of their work revealed that at 10 mg/L NOR and 60 W discharge power, the molality (mg/L) of oxidants O_3_, H_2_O_2_ and NO_3_^−^ progressively increased with the increase of plasma discharge time. However, the variation of solution pH did not show a significant influence on the production of ROS. Relatively, the authors showed that a higher removal rate percentage of NOR above 98% was achieved after 4 min of plasma exposure. The carcinogenic behaviour of NOR degradation intermediate by-products defined by TOC analysis was reduced at prolonged treatment time until their complete transformation to water, dissolved CO_2_ and harmless inorganics were achieved. These applausive results show the superiority of the DBD system in the battement of pharmaceutical toxins in water sources and should highly be considered as promising advanced technology in water and wastewater remediation.

**Figure 13:**
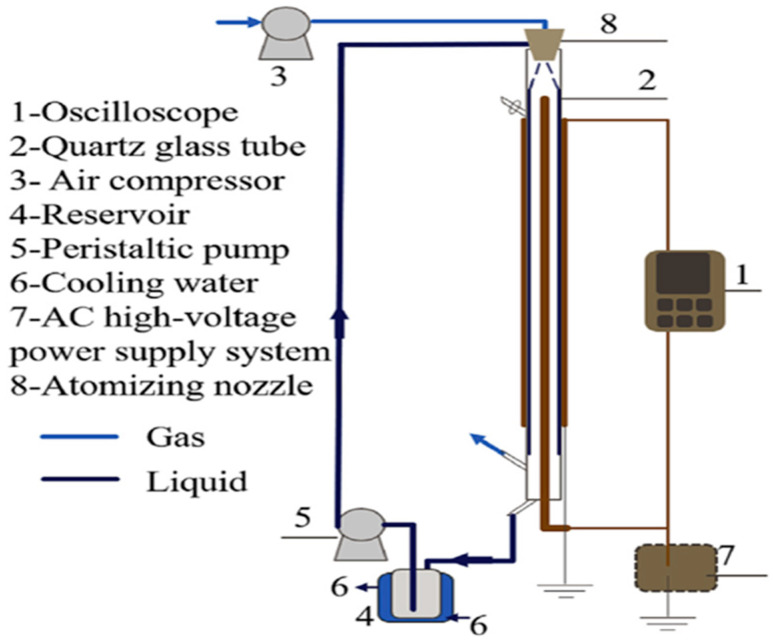
Spray dielectric barrier discharge (DBD) reactor set up used for the degradation of paracetamol [50].

An extended investigation on the influences of nitrite on paracetamol degradation in dielectric barrier discharge reactor shown in Figure 13 was conducted by Pan and Qiao [50].

At the following experimental conditions: discharge power 500 W, airflow rate 50 mL/min, paracetamol concentration varied from 10 mg/L to 100 mg/L, the authors noticed that the removal percentage of paracetamol decreased with the increase of initial concentration. That is, 99.99% removal of 10 mg/L paracetamol was achieved after 30 min compared to 53.3% removal reached with 100 mg/L at the same conditions. Besides, Pan and Qiao [50] observed that the degradation of paracetamol in their DBD system was quicker than that of its by-products that were slower due to their recalcitrant behaviour as dictated by TOC analysis. Fortunately, due to the robust capability of DBD to generate diverse oxidants, refractory degradation intermediates of paracetamol were oxidised to CO_2_, H_2_O, and simpler inorganics after prolonged treatment time, though their oxidation mechanisms were not elucidated. Several studies focusing on the use of DBD systems in the decomposition of pharmaceutical-based organic compounds and examination of discharge parameters have been established [168,169,170,171,172,173,174].

Most of these successful studies on the use of DBD technologies for the removal of pharmaceuticals show that DBD systems are robust and promising treatment techniques for the complete removal of pharmaceutical toxins from water sources. Though using different configurations, the authors of these studies have achieved excellent oxidation percentages above 80% of pharmaceutical decomposition by DBD alone. In some studies, the DBD configurations resulted in the total mineralisation of model pharmaceuticals, and their recalcitrant degradation intermediates by-products while others still require full revision of their electrode geometries. During these studies, this review points out that optimisation of various parameters including discharge power, solution pH, pollutant initial concentration, gas flow rate, plasma treatment time, is a crucial step in achieving high removal efficiencies of pharmaceutical toxins. Some of the authors in the studies reviewed above have successfully engaged in the determination of toxicity behaviour of the selected pollutants and their metabolites mostly followed by TOC analysis. While others still need to provide lots of effort to achieve these steps. On top of TOC analysis, this review suggests that environmental Researchers should conduct succinct toxicity tests before and after the plasma treatment of pharmaceutical contaminated water. This could also be an excellent indicator of the efficiency of plasma technologies used in the detoxification of pharmaceuticals from water and wastewater streams.

Degradation studies of pharmaceuticals using single dielectric barrier discharge (DBDs) systems are summarised in Table 3 and their extended efficiencies in Table 4. From the research investigations reviewed above, it could be observed that different degradation and mineralisation efficiencies of pharmaceutical compounds were achieved in these studies due to various oxidative species produced in the corresponding configurations. Thus, during the treatment of pharmaceuticals from water effluents, it is necessary to optimise parameters to achieve maximum desired removals. The highest removal efficiencies of most pharmaceuticals surpassed 90% as shown in Table 3 demonstrates that non-thermal plasma technologies mainly DBD configurations can still be considered as promising powerful methods for water and wastewater treatment. Likewise, the energy yields for the remediation of pharmaceuticals in polluted water is another effective factor that can be calculated to extensively assess the performance of DBD configurations summarised in Table 4.

A comprehensive comparison of non-thermal plasma reactors (corona discharges) in terms of energy yield was carried out by Malik [180] who showed that the energy yield required to decompose half concentration of the pollutant (G_50_) is an excellent factor to evaluate the performance of a typical plasma reactor. The energy efficiency (yield) of plasma configurations depends on various factors including the recalcitrance behaviour of intermediate by-products because of their competitive reactions occurring between themselves or with their parent compounds. Extended parameters such as type of plasma reactor, chemical structure, and concentration of pollutants, pH and additives, to mention only a few, may also impact the energy yields of plasma configurations [180]. Yet, Mok et al. [181] reported that the efficiency of AC powered dielectric barrier discharges generating UV, O_3,_ and co-species for the treatment of polluted water is 20 times higher than that of the reference actuator. In case air is used as the feed gas, Grabowski et al. [182] sustained that this might increase up to 400 times higher than the reference reactor when low concentrated solutions are remediated. Likewise, Nakagawa et al. [183] endorsed that the energy yield could further increase to 2000 times higher than the reference configuration when contaminated water is sprayed into pulsed-DBD in an oxygen-filled wire-to-cylinder electrode gap. So, in this review, the performance of various DBD configurations upon the degradation of pharmaceutical residues summarised in Table 4 was also evaluated by comparing their energy yield (G_50_) as suggested by Malik [180]. The data in Table 4 showed that the energy yield required for the degradation of pharmaceutical pollutants by DBD reactors varied from 0.17 to 7213.23 g/kWh. In this regard, 30 µg/L atrazine treated in an atmospheric pulsed DBD reactor at an input power of 1.7 W, only took 3.31 min to reach half of its concentration with an energy yield of 0.17 g/kWh, however, its total degradation required 45 min. On the other hand, 100 mg/L amoxicillin treated in DBD with falling liquid film reactor took less than a minute to reach half of its initial concertation at an energy yield of 7213.23 g/kWh while its complete decomposition was achieved within 10 min. These signify that the nature of the pollutant, the resistance of the degradation intermediate by-products as well as reactor electrode geometry impact upon the energy yield of DBD systems. Extensive scenarios could also be depicted in Table 4. For instance, in Table 4, the following pharmaceuticals, sulfadiazine antibiotics, enalapril, carbamazepine, and ampicillin treated in the same DBD with falling liquid film reactor at different concentrations and input powers resulted in different energy yields in the order of 19.60, 34.6, 310.40 and 2379.30, respectively. This shows that the type of pollutant, concentration, and power applied for their decomposition affect their energy yields as well [180]. Next, ampicillin, oxacillin, and pentoxifylline treated with the same liquid film falling DBD reactor at the same concentration (100 mg/L) but different input powers resulted in different energy yields (2379.30, 2381.12 and 2208.03, correspondingly) and half-life times (1.80, 1.80 and 16.5 min, respectively). This confirms that even when selected at the same amount, the nature of the pollutants plays a crucial role in the determination of energy yield and hence the reactor efficacy. Their difference in half-life times indicates that pentoxifylline was the most recalcitrant and required 60 min for full degradation as compared to ampicillin and oxacillin that were removed after 30 min of plasma discharge experiment (Table 4). All these scenarios show that DBD technologies are effective methods for water and wastewater remediation. However, the choice of a DBD configuration may depend not only on degradation efficiencies, half-life time but also on their energy yield that is required to reach half of their initial concentrations.

**Figure 14:**
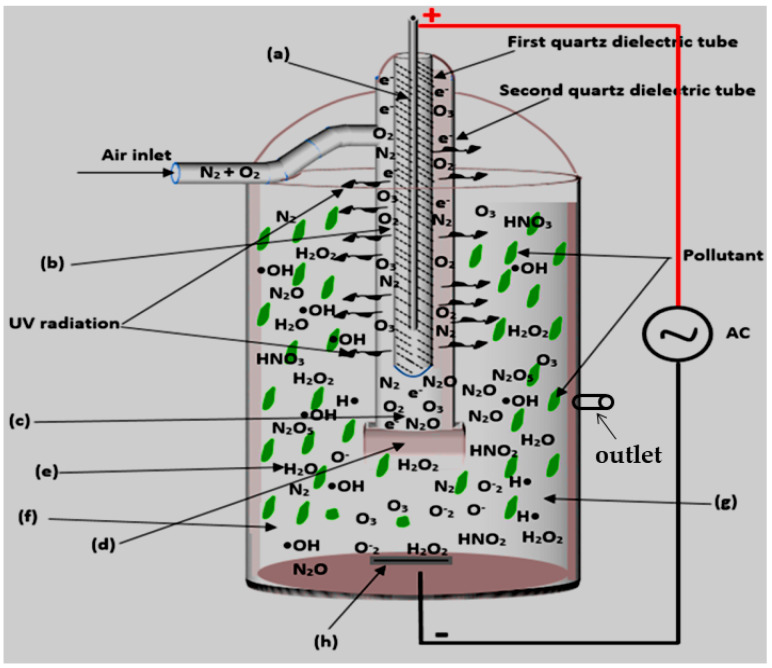
Different reaction zones encountered in the dielectric barrier discharge (DBD) system [120].

Extensive reconfigurations of DBD reactors and their subsequent optimisation could be convenient suggestions to overcome most of the limitations previously highlighted and hence cognitive means for total oxidation of recalcitrant pharmaceutical compounds. From the DBD configurations employed in the degradation of pharmaceuticals as reviewed in this text, there is little information on the use of double cylindrical dielectric barrier discharge (DCDBD) configuration (Figure 14) for the removal of pharmaceuticals.

The DBD has proven to be a potent configuration for the decomposition of organic compounds in aqueous media, hence this particular configuration could be applied for the effective degradation of pharmaceutical pollutants [20,120]. Therefore, this review advocates the use of DCDBD as a more powerful AOP as compared to previous AOP techniques. DCDBD delivers enormous benefits such as great reactor stability and efficiency, uniformity of the UV-vis, energy saving, rapid processing, use of no chemicals as well as the non-destructive impact on the ecosystem. This system is also capable of generating ozone, hydrogen peroxide, singlet oxygen, superoxide radicals, hydroxyl radicals, and other active species. The combination of these reactive species has been reported to degrade biological and chemical pollutants rapidly and efficiently. The DCDBD also affords great degradation efficiencies of targeted pollutants [43,157,184].

##### Double Cylindrical Dielectric Barrier Discharge System—Formation of the Free Active Species in Different Regions

A choice of a double cylindrical DBD plasma reactor (schematically shown in Figure 14) as a significant AOPs is based on its ability to decompose persistent organics and eliminate microbes. The system is also capable of generating ozone, hydrogen peroxide, singlet oxygen, superoxide radicals, hydroxyl radicals, and other active species. The combination of these reactive species has been reported to degrade biological and chemical pollutants rapidly and efficiently. To understand the prominence of DBD systems and where the UV light and some of the highlighted species are formed, the different reaction zones have subsequently been discussed.

**Region (a)**

In this region, electric discharge is a source of highly energized particles (electrons) whose motion gives rise to electric and magnetic fields. The electric field accelerates the particles while the magnetic field facilitates their deviation along the anode electrode and its curvature. Kogelschatz [153,154] reported the continuous flow of current increases electron density and their motion around the anode rod sometimes leads to their collision, often referred to as electron avalanche. This magnifies the intensity of the electric and magnetic fields around the high voltage anode electrode. Locke and Shih [185] supported that the use of the dielectric material (inner quartz dielectric tube) as an insulator of the anode does not only minimize the number of charges transported by a single micro discharge (microplasma) but also permits the even distribution of the micro discharges around the anode surface area. So, the anode rod and the inner dielectric quartz tube together constitute one single power electrode that becomes a permanent source of high energy electrons.

**Region (b)**

Zone (b) also called discharge/air gap, is the space between the two dielectrics (inner and outer quartz dielectrics tubes). In this region, the feeding gas (dry air from an air pump) is passed through and interacts with the highly powered anode electrode. Since air mostly consists of oxygen (O_2_) and nitrogen (N_2_), these molecules readily react with high energy electrons, generating not only UV light but also leading to the production of various types of oxygen and nitrogen-based species atomic oxygen, ozone, peroxide ions formed via dissociation, ionization, recombination and associative chemical reactions [85].

**Region (c) and (d)**

Region C is also part of the discharge zone. This is the zone where oxygen, nitrogen, and the resulting species produced in the plasma region are circulated through region C until they reach the outlet (zone D) of the double cylindrical DBD plasma reactor. At zone C, molecular and ionic species such as O_3_, O_2_, O_2_^−^, N_2_ and N_2_O generated in regions B and C are bubbled into the bulk solution to induce oxidation of the target pollutant [185]. In the DBD electrode configurations, zone C might sometimes represent the sealing area of the inner tube. This is usually used to protect the bottom of the inner tube against the pressure resulting from high voltage discharges. Nevertheless, where there was no sealing in the DBD plasma reactor, a reasonable voltage could be applied for DBD experiments.

**Region (h)**

In this region of the bulk solution, a magnetic stirring bar with stirring speed in the range 50–60 rpm could be used to evenly disperse the reactive and active species from zone D into the contaminated solution.

**Region (e)**

Zone (E) represents the region next to the outer tube (second dielectric quartz tube). In this area of the DBD system, the UV radiations diffusing in the bulk solution dissociate water molecules into OH radicals. Therefore, the decomposition of H_2_O molecules by UV light is suggested by [28]. 

In addition to this, the dissolved O_3_ mostly in region E can also be irradiated by the UV light [186] and consequently decomposed into H_2_O_2_. Furthermore, the shining UV also illuminates H_2_O_2_ and dissociates it into OH radicals which non-selectively attack and mineralise the target contaminant into the water and dissolved CO_2_ [187].

**Regions (f) and (g)**

In these regions, the active species such as ozone and OH radicals, diffuse in the bulk solution and destroy the pollutant. Moreover, in regions F and G, the free reactive species such as hydroxyl radicals resulting from various dissociation processes by UV light might recombine to form hydrogen peroxide [186].

Additionally, various other species such as reactive nitrogen-based species (RNS) including NOx, formed via several chemical reaction chains are also found in these DBD zones. The mechanism of formation of NOx species in the DBD plasma system had earlier been proposed and when the plasma fluid generated in zone B is transferred to the bulk solution, the NO_2_ coexisting with ozone is probably dissolved to form nitric acid and nitrous acid [187].

Based on this descriptive discussion, DBD reactor configurations have shown great effectiveness in the removal of pharmaceutical contaminants with more than 90% of pharmaceuticals removal efficiencies recorded. Though various limitations were encountered during DBD plasma experiments, these could be alleviated by reconfiguring DBD electrode geometries and their optimization parameters. Among common DBD configurations summarised by Kogelschatz et al. [154], only a few or no authors have explored double cylindrical configurations for the decomposition of pharmaceutical compounds from water and wastewater. For instance, Rong et al. [157] used a double cylindrical DBD configuration for the decomposition of a nonsteroidal anti-inflammatory drug (NSAID) diclofenac in an aqueous solution and the results obtained showed an input power of 50 W and pH of 6.1 a 10 mg/L diclofenac was oxidised within 10 min of the DBD experiment. These authors claimed that the addition of Fe^2+^ in the liquid phase promoted the oxidation of diclofenac drug. A similar investigation using double cylindrical DBD was recently conducted by Tijani et al. [184] for the decomposition of a 2-Nitrophenol water pollutant and absolute removal efficiency was achieved. Apart from these mentioned studies, the use of double DBD reactors for the degradation of pharmaceuticals has scarcely been documented. Therefore, this review proposes that double cylindrical DBD electrode geometry could efficiently be employed to decompose pharmaceutical residues from water and wastewater. Besides double cylindrical DBD configuration, compatible excilamp DBD technologies have recently been claimed effective for water and wastewater decontamination [188,189,190,191,192].

##### DBD Driven Excilamps as a Possible Candidate to Decompose Pharmaceutical Compounds

The dielectric barrier discharge excilamp (DBD excilamp or DBD driven excilamps) is one more technology for the efficient production of ultraviolet (UV) and vacuum ultraviolet (VUV) radiation and photochemical water pollutants mineralisation [154,192,193,194]. In general, excilamps are a class of spontaneous radiation sources based on transitions of rare gas excited dimers R_2_*, on halogen excited dimers X_2_*, or on rare-gas halide excited complexes RX* [189,191]. The most attractive for use in practice today are excilamps on Xe_2_*, KrCl*, XeCl* and XeBr* molecules, which produces radiation flux in a relatively narrow spectral region with maximum wavelength at 172, 222, 308 and 282 nm, correspondingly. This ensures the selectivity of various photochemical reactions. An important feature of excimer lamps is currently the absence of mercury in the bulb. Instantaneous ignition of excilamps implies no warm-up time and immediate availability to work. Today is clear that DBD excilamps are commercially attractive.

**Figure 15:**
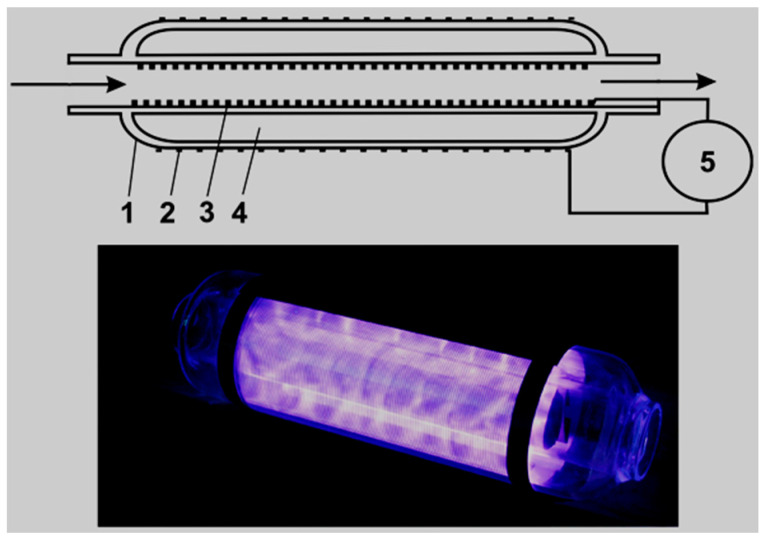
Coaxial excilamp design (**top**) and external view during operation (**bottom**): 1—excilamp bulb; 2—external perforated electrode; 3—internal perforated electrode; 4—discharge gap; 5—high-frequency voltage generator.

DBD excilamps have diversity in the design concept but the most interest in photochemistry applications is so-called coaxial geometry, which is shown in Figure 15. In this case, DBD excites the operating gas or gas mixture in the spacing between two coaxial quartz tubes. The external and internal electrodes are connected to a high-frequency voltage generator (with voltage amplitudes up to several kV). The pulse generator guarantees radiation dosage. Discharge ignition leads to the formation of exciplex or excimer radiation.

The internal cavity of the DBD excilamp can be used for pumping aqueous solutions containing contaminants (as indicated by the arrows in Figure 15). The radiation of exciplex molecules provides photo mineralisation of dissolved organic matters.

Photomineralisation can be generally expressed as C_n_H_m_Y_z_ (*hv*, O_2_) → nCO_2_ + (m–z)/_2_ H_2_O + zHY, where Y are heteroatomic organic molecules that are transformed into the corresponding mineral acid HY (HNO_2_, H_2_SO_4_, HCl, HNO_3_, etc.). A similar reaction proceeds during the interaction of an oxidiser and UV radiation with organometallic complexes, with metal ions being additionally released into the solution. Decomposition of dissolved pollutants such as pharmaceuticals is possible with no special oxidisers (so-called, reagentless photolysis) or with ecologically clean oxidisers (e.g., hydrogen peroxide).

Although photo mineralisation is realisable in various ways, it involves an efficient generation of OH^.^, radicals almost without exception. These radicals have a high oxidising ability and a short lifetime, react with all matters and can be obtained by different methods. Besides OH**^.^** radicals, oxidation can be realised by superoxide anions (OH_2_**^.^**^–^) and O_3_ particles and singlet oxygen (O**^.^**). It was conventionally proven that the most valuable range for direct photolysis of dissolved organic matters is 200–280 nm, because most components (dissolved organic and inorganic) contained in water absorb radiation in this range.

**Figure 16:**
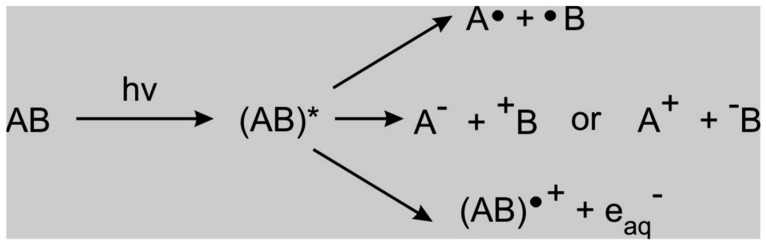
Possible scenarios of events indirect photolysis.

Direct photolysis causes excitation of singlet states of organic matters with their subsequent relaxation to triplet states. These excited states can then be exposed to homolysis, heterolysis and photoionisation (Figure 16). For direct photolysis, for example, the DBD excilamps on molecules KrCl* (222 nm), XeBr* (283 nm) and Cl_2_* (259 nm) can be used [195]. Here the coaxial excilamps (Figure 12) with the pumping of solution through their inner cavity are applied. That is, the lamp itself is a photoreactor (flow-through photoreactors) [196] and the quality of lamps can be one or more [197].

A quite interesting wavelength range is 100–200 nm. The concentration of matters dissolved in water is normally low. Therefore, VUV radiation is absorbed mainly by water whose concentration is 55.5 M; that is in typical conditions, the amount of water is a million times larger than that of matter dissolved in it and hence the radiation energy of a VUV source is expended mainly in water homolysis H_2_O + *hv* → H_2_O* → H**^.^** + OH**^.^** [133,167]. Next, the pollutant/pharmaceutical reacts with formed radicals and is mineralised. This is the so-called VUV photolysis. The advantages of VUV photolysis are the following: (1) extremely high concentration of hydroxyl radicals (locally); (2) no need for additional oxidisers (in situ generation of H_2_O_2_ and O_3_); (3) efficient photo mineralisation of organics in a solution; (4) reduced effect of bicarbonates and nitrate ions on the photolysis rate.

Numerous examples of dissolved organic matter photolysis are given in [192,198]. Analysis of available data shows that the most efficient excilamp for AOPs is an excilamp on Xe_2_* dimers (λ~172 nm). The shortcoming of VUV excilamps is the small depth of VUV penetration into water. The contradiction here is the following: when exposed to VUV irradiation, water is bound to be a solvent but its presence in an irradiated solution is undesirable because water decreases the effective volume of the process and impedes the interaction of radiation with dissolved organic matters.

This contradiction can be resolved in several ways. It is possible to use photolysis with water in a different aggregate state like, in Oppenländer and Fradl, [199] where wastewater was primarily transformed into vapour and was then irradiated by a Xe_2_-excilamp. This greatly enlarged the reaction volume and accelerated the process of phenol photolysis. The process was termed photoreactive distillation. It is possible to follow a different way, i.e., to find chemical compositions in which the water content is small, but the effect of this small amount is quite significant.

**Figure 17:**
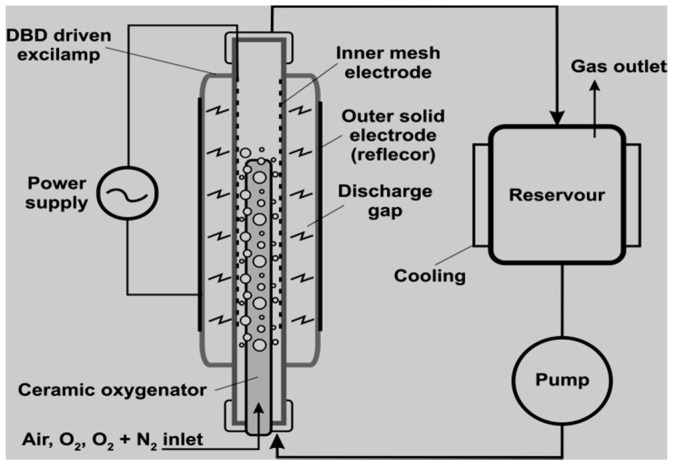
Schematic drawing of set-up with dielectric barrier discharge (DBD) driven Xe_2_-excilamp for UVU photolysis [200].

Finally, the problem can be solved with the use of hydrodynamics effects, for instance by applying static mixers or higher flow rates, in other words, by enhancement of mixing of the solution. The most simple and effective way to increase the UVU photolysis rate is the injection of O_2_ (or air) into DBD driven excimer coaxial flow-through photoreactor [200]. This reactor system is presented in Figure 17. It has been shown that the rate of mineralisation of several organic substances other than pharmaceuticals (1-heptanol, benzoic acid and potassium hydrogen phthalate) dissolved in water was enhanced significantly by oxygen or air injection.

This was compared to conditions under nitrogen injection or without gassing. The authors concluded that the great potential of this technique is related to the fact that the geometry of Xe_2_-excilamps is widely variable. Moreover, small or large DBD driven excilamp reactors with incorporated tubes and aerators of different sizes and materials are possible. Also, this technology is comparatively simple.

So far, the above techniques of using DBD excilamps have not yet found a wide application in the pharmaceutical pollutant destruction. Although Oppenländer et al. [200] designed the first flow-through photoreactors precisely for the task of studying the photostability of drugs [196]. We believe that DBD excilamps are undervalued techniques and have great potential for destroying water pollutants such as pharmaceutical toxicants.

Therefore, unlike other AOPs, this technique allows better control of physical factors when exposed to water. This means that it is possible to obtain stable results of photolysis, which is important for the introduction of technology in the water treatment industry. Moreover, flow reactors can treat relatively large solution volumes, which is also important. Therefore, we believe that research using DBD excilamps should continue.

#### 3.2.4. Summary of the Degradation of Pharmaceutical Compounds by DBDs

In comparison, the dielectric barrier discharges described in this paper show that all configurations converge towards the mineralisation of targeted pharmaceutical pollutants. These electrode geometries produce not only UV light, but they are also sources of various oxygen-based species that participate directly or indirectly in the generation of non-selective powerful oxidant hydroxyl radicals (OH**^.^**), that oxidise and mineralise pharmaceutical toxins and certainly other organic micropollutants ideally into dissolving CO_2_, H_2_O, and harmless inorganic by-products.

In these studies, reasonable degradation efficiencies of pharmaceutical compounds were achieved [155,156,158,159,160,161]. Even though pharmaceutical compounds have been decomposed using various DBDs, some DBD configurations present several limitations. For example, the pollutant concentration, pH, kinetics, and energy yield efficiency required in the process are very important parameters that influence contaminant removal; however, most of these parameters in the decomposition of pharmaceuticals from water and wastewater were not fully examined in most publications reviewed. A complete investigation of these factors is required to establish the best degradation conditions for the complete decomposition of these contaminants. Their concentration is a key parameter since the decomposition of pharmaceuticals decreases with an increase of pollutant concentration [113]. This is probably associated with the chemical stability of their molecular structures designed to resist types of oxidants. Moreover, pharmaceuticals decompose at different pH values, some have been well removed in acidic, neutral, or basic media [155]. Additionally, the stability of these compounds may also slow down the rate of their degradation which usually leads to pseudo-first-order kinetics [201]. Analysis of the studies reviewed in this paper also shows that the use of UV and ozone generators requires a certain amount of energy to induce oxidation processes.

However, most papers reviewed in this text did not investigate the energy yield /energy consumption that dictates the efficiency of the AOPs. With a lower energy consumption of about 1 J/s, double cylindrical DBD and DBD excilamp on Xe_2_* dimers (λ~172 nm) could also be used not only as efficient energy-saving methods that produce UV, O_3_, H_2_O_2_, OH**^.^** but also, as promising novel technologies for the oxidation of pharmaceutical compounds from water effluents [20,200].

Moreover, the limited use of chemicals in double cylindrical DBD and Xe_2_-excilamp configurations is beneficial to avoid more toxicity of the effluent being treated. Various studies have demonstrated that degradation metabolites are sometimes more toxic than parent pollutants and the combination of various homogeneous AOPs such as UV/O_3_, O_3_/H_2_O_2_, UV/ H_2_O_2_ and UV/O_3_/H_2_O_2_ have been proved to largely generate powerful non-selective hydroxyl radicals through side reactions [28,202]. Though various (planar) DBDs reactor configurations have been used to decompose organic pharmaceutical pollutants from water and wastewater [48,121], double cylindrical and Xe_2_-excilamp DBDs have not been widely used in the decomposition of pharmaceuticals from water effluents. In the single DBD reactor configuration, the high voltage electrode is protected by one dielectric barrier and is usually exposed to feeding gas thus the exposure of the high voltage electrode to a circulating gas could result in its corrosion [20,48,203].

Likewise, in double cylindrical and Xe_2_-excilamp DBD configurations, the high voltage electrode is protected by two dielectric barriers and separated from feeding gas which in turn prevents corrosion and facilitates long-term experimental runs. The region between the two dielectrics represents the plasma zone in which various oxidative species are continuously produced and directly circulated into the polluted solution.

This review thus emphasises that though single DBD systems have been used for water and wastewater treatment, some configurations still show limitations that need to be overcome by revising and optimising DBD electrode geometries. Hence, double cylindrical and Xe_2_-excilamp DBD for instance could be robust technological alternatives for the degradation of targeted pharmaceuticals.

A variety of degradation/decomposition intermediates and mechanistic pathways should be detected and quantified to eliminate the challenge of completely removing pharmaceuticals due to the recurrent phenomenon of most persistent organic pollutants degradation intermediates often recombining to form large complexes. The successful prediction of pharmaceuticals degradation mechanistic pathways to promote complete removal in final treated effluents using configurations such as double cylindrical and Xe_2_-excilamp DBDs are essential to achieve integrated water treatment processes free from all forms of xenobiotics.

## 4. Conclusions

Various categories of pharmaceutical toxins are abundantly present in aquatic environments across the globe and pharmaceutical industries remain their principal sources. The predominance of pharmaceutical abundance across the globe is because environmental pollution and regulation awareness is more advanced in some areas (Asia, North and Central America, South America, Europe) as compared to Africa. This review paper presents studies that prove that single and double dielectric barrier discharges (BDs) successfully remove pharmaceuticals from water and wastewater. The removal efficiency can be improved by revision of electrode geometries and optimisation of reactor configurations as desired. 

The DBD reactors were proved to have numerous advantages such as the production of UV light and various oxygen-based species. Based on these benefits, dielectric barrier discharges (DBDs) are potential energy-saving, cost-effective, and environmentally benign wastewater treatment technologies. The studies reviewed in this paper showed that pollutant concentration, pH, kinetics and energy required in the oxidation processes are essential parameters that determine the decomposition of pharmaceutical compounds from water effluents.

Proper optimisation of some specific parameters for particular organic pharmaceutical pollutants removal as well as the degradation intermediates and degradation pathways of the pollutants were not fully carried out/not provided in most publications reviewed, hence a complete optimisation study of these parameters during degradation of pharmaceutical compounds is crucial. The identification and determination of degradation pathways are of great importance and require further investigation. Finally, toxicity tests of the identified intermediates and quantification of free active species generated during the oxidation process also demand adequate research attention.

Furthermore, we also believe that another DBD device—excilamps—should be used in future studies. DBD review demonstrates that this technique has several advantages and its potential for the decomposition of pharmaceutical compounds is underutilized.

## Figures and Tables

**Table 1:** Global occurrence of pharmaceutical compounds in water and wastewater.

**Africa**	**Countries**	**Types of Pharmaceuticals**	**Pharmaceutical Compounds**	**Concentration**	**Sources**	**References**
	South Africa, (Kwazulu-Natal)	Antipyretics, antibiotics, lipid regulators, antipsychotic anti-epileptic drug	Aspirin nalidixic acid, caffeine, bezafibrate, clozapine	0.82–118 mg/L	Msunduzi river, wastewater	[68,69]
	Enugu, South-East Nigeria	Antibiotics, oestrogens, lipid-lowering drugs, non-steroidal anti-inflammatory drugs	Diclofenac	8.84–1100 µg/L	Wastewater treatment plants	[67]
	Chirapatre Estates in Kumasi, Ghana		Paracetamol	NA	Irrigation water	[66]
**Asia**	South China	PPCPs, synthetic steroid oestrogens, endocrine-disrupting phenols, acidic pharmaceuticals	Nonylphenol, bisphenol A, triclosan, 2-phenyl phenol, methylparaben, propylparaben, salicylic acid, clofibric acid, Ibuprofen	65 ng/L–1417 ng/L	Urbane rivers of the Pearl River delta	[79]
	China (Beijing)	Antibiotics, antilipidemic, anti-inflammatory, antihypertensive, anticonvulsant, stimulant, insect repellent, antipsychotic	Caffeine, N, N-Diethyl-meta-toluamide (DEET)	2.2–320 ng/L	WWTPs	[80]
	China	Antibiotics, anti-inflammatories, lipid regulators	Carbamazepine, caffeine, N, N-diethyl –m-toluamide	BLD-6 to 35.31 ng/L	Groundwater	[81]
	Yang pu district, China	Antibiotics, analgesics, antiepileptics, lipid regulators	Caffein, paracetamol, propranolol, azithromycin, clarithromycin	100–857 ng/L	Urban rivers	[82]
	Japan	Veterinary drugs and antibiotics	Amantadine, carbamazepine, epinastine, ibuprofen, iopamidol, oseltamivir acid	NA	Finished drinking water	[83]
	India	Antipsychoactive, antihypertensive, antimicrobials, analgesics, stimulants	Carbamazepine, atenolol, triclocarban, caffeine, ciprofloxacin, enoxacin, ketoprofen, erythromycin, naproxen, diclofenac, enrofloxacin	NA	Conventional activated sludge WWTPs, hospital WTPs, rivers, and groundwater.	[63]
	South Korea	Antibiotics, hormones, antihypertensive, stimulants	Acetaminophen, atenolol, lincomycin, hormones, antihypertensive, antiepileptic	˃10 µg/L	WWTPs, sewage effluent	[84]
	Malaysia	NSAIDs, stimulants	Caffeine and diclofenac	Non-detected-54 ng/L	Langat and Muar Rivers	[85]
**North & Central America**	**Countries**	**Types of pharmaceuticals**	**Pharmaceutical compounds**	**Concentration**	**Sources**	**References**
	Mexico	Antibiotics, non-steroidal anti-inflammatory drugs	Acetaminophen, atenolol, carbamazepine, clarithromycin, diclofenac, ibuprofen	0.01–6800 ng/L	Sewage effluent, aquaculture	[86]
	USA, Pennsylvania	Pharmaceutical compounds, hormones, organic wastewater compounds (OWCs)	Acetaminophen, caffeine, carbamazepine, tylosin, sulfadimethoxine, sulfamethoxazole, oxytetracycline	24–1340 ng/L	Pennsylvania Waters	[64]
	USA, Georgia	Pharmaceuticals and personal products (PPCPs): Antibiotics, analgesics, anti-inflammatory, diuretics, antipyretics, antihypertension, fragrances, flame retardants, disinfectants, antiseptics, and pesticides	Acetylsalicylic acid, 17-Ethinylestradiol, dimethylaminophenazone, hydrocodone, Cashmeran, biphenylol, Tetrabromobisphenol A	0.0002–230 µg/L	Wastewater treatment plant (WWTP) influents	[87,88]
	USA, North Carolina	Pharmaceuticals: Antibiotics, analgesics, diuretics, antipyretics, anti-inflammatory, antihypertension	Acetaminophen, caffeine, tylosin, carbamazepine, sulfadimethoxine, sulfamethoxazole, oxytetracycline,	˃ppb	Wastewater treatment plant (WWTP) effluents	[89]
	USA, Liberty Bay, Puget Sound, Washington	Herbicides and pharmaceutical and personal care products (PPCPs) and flame retardants	N, N-diethyl-meta-toluamide, caffeine, and mecoprop	NA	Surface Water and Groundwater in WWTPs	[90]
	USA, Southern Nevada	Contaminants of emerging concern	Naproxen, gemfibrozil, atenolol	˃1 μg/L	Municipal wastewater effluents and marine receiving water	[65]
**South America**	Argentina	Analgesics, anti-inflammatories anticonvulsant, Stimulants	Carbamazepine, caffeine and ibuprofen	0.03–4.2 μg/L	Wastewater effluents	[91]
	Brazil	Lipid regulators, anti-inflammatories	Bezafibrate, gemfibrozil, ibuprofen, diclofenac	0.1 to 0.5 μg/L	Effluents of sewage treatment plants (STPs)	[92]
	Colombia	Antibiotics, anticonvulsants, non-steroidal anti-inflammatory drugs	Acetaminophen paracetamol, carbamazepine diclofenac, ibuprofen	NA	Wastewaters effluent and influent surface waters	[93]
**Europe**	**Countries**	**Types of pharmaceuticals**	**Pharmaceutical compounds**	**Concentration**	**Sources**	**References**
	Finland	Antibiotics, antiepileptic, anti-inflammatories	Ciprofloxacin, norfloxacin and ofloxacin carbamazepine diclofenac, ibuprofen, ketoprofen, naproxen	NA	Sewage Treatment Plants effluents, Surface Waters	[94]
	France	Psychostimulants, non-steroidal anti-inflammatory drugs, iodinated contrast media, and anxiolytic drugs	Ciprofloxacin, norfloxacin and ofloxacin carbamazepine diclofenac, ibuprofen, ketoprofen, naproxen	NA	Surface water	[55]
	Germany	Antibiotics	Penicillins, carbamazepine, clofibric acid, iopromide	NA	Sewage treatment plant (STP) discharges, groundwater, Wastewater and household waste	[95]
	Italy	Antibiotics, anti-inflammatory	Propranolol, paracetamol	0.001 to 284 ug/L	NA	[60]
	Netherland	Antibiotics, anticonvulsants	Carbamazepine, erythromycin	0.31–0.90 ug/L	Sewage Treatment Plants effluents, surface water	[96]
	Portugal	Antibiotics, anticonvulsants, anti-inflammatory	Ibuprofen, sulfamethoxazole, ketoprofen, carbamazepine, fluoxetine	NA	Influents and effluents of two wastewater treatment plants	[97]
	Romania	Antibiotics, anticonvulsants, anti-inflammatory	Sulfamethoxazole, diclofenac, carbamazepine, trimethoprim, thiabendazole, clotrimazole	5 to 50 ng/ L	Rivers	[98]
	Serbia	Antibiotics, anticonvulsants, anti-inflammatory	Sulfamethoxazole, carbamazepine, trimethoprim	NA	River sediments, surface, and groundwater	[62]
	Spain	Anti-inflammatories, antidepressant	Ketoprofen, naproxen, fluoxetine	0.3–324.7 ng/L	Wastewater treatment plants discharges	[99]
	Switzerland	Antibiotics, anti-inflammatories	Diclofenac, carbamazepine	NA	Wastewater, groundwater	[100]
	Turkey	Antibiotics, anticonvulsants, anti-inflammatory	Acetaminophen, carbamazepine, hospital wastewater	up to 160 µg/L	Sewage-treatment plant discharges	[61]
	United Kingdom	Sewage treatment plants and wastewater	Ibuprofen and ciprofloxacin	NA	Sewage treatment plant (STP) discharges, Groundwater	[101]

**Table 2:** Pharmaceuticals commonly found in the aquatic environment [113].

Penicillin-Type Antibiotics	Anti-Inflammatory Drugs /NSAIDS	Beta-Blockers/ Antihypertensive	Steroids and Hormones	Lipid Regulators	Anticonvulsants/Antiepileptic	Antidepressants	Cancer Therapeutics
Amoxicillin	Acetaminophen	Atenolol	Diethylstibestrol	Bezafibrate	Carbamazepine	Diazepam	Cyclophosphamide
Ampicillin	Aspirin (acetylsalicylic acid)	Betaxolol	Diethylstibestrol acetate	Clofibric acid	Dilantin	Furosemide	Ifosfamide
Chlortetracycline	Diclofenac	Diltiazem	17-α-ethinyl estradiol	Fenofibrate	Phenobarbital	Fluoxetine	
Ciprofloxacin	Codeine	Enalapril	17-β-estradiol estrone	Gemfibrozil	Primidone	Meprobamate	
Erythromycin	Ibuprofen	Furosemide	Estrone			Paroxetine	
Flumequine	Indomethacin	Losartan	Mestranol				
Lincomycin	Metamizole	Metoprolol					
Penicillin	Naproxen	Nadolol					
Ofloxacin	Phenazone	Propranolol					
Oxacillin		Sotalol					
Oxytetracycline							
Spiramycin							
Streptomycin							
Sulfamethoxazole							
Trimethoprim							

**Table 3:** Degradation of pharmaceutical compounds by various dielectric barrier discharge configurations.

Pharmaceutical Compounds	Wastewater Source	Initial Concentration (mg/L)	pH	DBD Reactor Configuration and Process Design	Treatment Scale	Treatment Time (min)	Degradation %	Energy (mol/J)	Intermediates	References
sulfadiazine antibiotics	Synthetic wastewater	10	9	Water falling film DBD	Laboratory scale	30	87	38.6 × 10^−12^	Yes	[157]
Veterinary antibiotics	Synthetic wastewater	5	NA	Single cylindrical DBD plasma reactor	Laboratory scale	30	60–90	NA	Yes	[159]
carbamazepine	Synthetic wastewater	20	NA	Ex situ DBD system	Laboratory scale	60	81–90	NA	Yes	[156]
atrazine	Synthetic wastewater	0.03	5.06	Atmospheric pulsed DBD	Laboratory scale	45	61	NA	Yes	[175]
17b-estradiol	Synthetic wastewater	0.4	5.6	non-thermal plasma (DBD)	Laboratory scale	30	72.0	NA	Yes	[176]
pentoxifylline	Synthetic wastewater	100	7	DBD coaxial reactor configuration	Laboratory scale	60	92.5	NA	Yes	[155]
Three b-lactam antibiotics	Synthetic wastewater	100	NA	DBD coaxial reactor configuration	Laboratory scale	10–30	25–30% of TC	NA	Yes	[158]
(Atrazine, chlorfenvinfos, 2,4-dibromophenol, and lindane)	Synthetic wastewater	1–5	NA	Batch reactor (R1) and Coaxial reactor (R2)	Laboratory scale	15	86.6–98.0 (R1) and 40.3–93.6 (R2)	NA	Yes	[177]
iopromide	Synthetic wastewater	17	NA	DBD	Laboratory scale	10	99	0.14 × 10^−9^	NA	[178]
enalapril	Synthetic wastewater	50	NA	DBD with falling liquid film	Laboratory scale	120	99.4	2 × 10^−9^	Yes	[179]
carbamazepine	Synthetic wastewater	20	NA	DBD with falling liquid film	Laboratory scale	60	90.7	0.18 × 10^−9^	Yes	[156]
carbamazepi	Synthetic wastewater	23.6	NA	DBD rotating drum reactor	Laboratory scale	60	94	52.2 × 10-12	NA	[162]
iopromide	Synthetic wastewater	79.1	NA	DBD rotating drum reactor	Laboratory scale	60	98	54.4 × 10^−12^		[162]
clofibric acid	Synthetic wastewater	21.5	NA	DBD rotating drum reactor	Laboratory scale	30	100	0.11 × 10^−9^		[162]
amoxicillin	Synthetic wastewater	100	NA	DBD with falling liquid film	Laboratory scale	10	100	79.8 × 10^−9^	Yes	[158]
ampicillin	Synthetic wastewater	100	NA	DBD with falling liquid film	Laboratory scale	30	100	23.1 × 10^−9^	Yes	[158]
oxacillin	Synthetic wastewater	100	NA	DBD with falling liquid film	Laboratory scale	30	100	18.7 × 10^−9^	Yes	[158]
pentoxifylline	Synthetic wastewater	100	NA	DBD with falling liquid film	Laboratory scale	60	92	16 × 10^−9^	yes	[155]
carbamazepine	Synthetic wastewater	23.6	NA	DBD	Laboratory scale	30	98	21.7 × 10^−12^		[161]
clofibric acid	Synthetic wastewater	21.5	NA	DBD	Laboratory scale	30	100	22.3 × 10^−12^	NA	[161]
iopromide	Synthetic wastewater	79.1	NA	DBD	Laboratory scale	30	99	22 × 10^−12^	NA	[161]

**Table 4:** Energy yield calculated at 50% degradation of pharmaceutical compounds by various dielectric barrier discharge configurations shown in Table 3.

Pharmaceutical Compounds	Initial Concentration (mg/L)	Treatment Time (min)	DBD Reactor Configuration and Process Design	Power (w)	Rate Constant k_r_ (min^−1^)	Half-Life Time t_1/2_ (min)	Energy Yield (G_50_) (g/KWh)	References
Sulfadiazine antibiotics	10	30	Water falling film DBD	150	6.8 × 10^−2^	10.19	19.60	[157]
Veterinary antibiotics	5	30	Single cylindrical DBD plasma reactor	8.9	3.05 ×10^−2^	22.72	12.16–12.14	[159]
Carbamazepine	20	60	Ex situ DBD system	0.7–12	2.76 × 10^−2^–3.8 × 10^−2^	25.10–18.23	308.70–18.01	[156]
Atrazine	0.03	45	Atmospheric pulsed DBD	1.7	2.09 × 10^−1^	3.31	0.17	[175]
17b-estradiol	0.4	30	Non-thermal plasma (DBD)	NA	4.24 × 10^−2^	16.34	NA	[176]
Pentoxifylline	100	60	DBD coaxial reactor configuration	1	4.32 × 10^−2^	16.04	2220.29	[155]
Three b-lactam antibiotics	100	10–30	DBD coaxial reactor configuration	2	9.7 × 10^−3^	71.44	1820.88–2206.49	[158]
(Atrazine, Chlorfenvinfos, 2,4-dibromophenol, and lindane)	1–5	15	Batch reactor (R1) and Coaxial reactor (R2)	30	1.34 × 10^−1^–2.6 ×10^−1^	5.17–2.66	2.741–12.95	[177]
Iopromide	17	10	DBD	NA	4.6 ×10^−1^	1.50	NA	[178]
Enalapril	50	120	DBD with falling liquid film	NA	4.26 × 10^−2^	16.26	34.6	[179]
Carbamazepine	20	60	DBD with falling liquid film	0.7–12	3.95 × 10^−2^	17.54	310.40–18.10	[156]
Carbamazepine	23.6	60	DBD rotating drum reactor	250–750	4.68 × 10^−2^	14.81	10.65–3.55	[162]
Iopromide	79.1	60	DBD rotating drum reactor	250–750	6.5 × 10^−2^	10.66	37.22–12.41	[162]
Clofibric acid	21.5	30	DBD rotating drum reactor	250–750	3.32 × 10^−1^	2.08	20.44–6.81	[162]
Amoxicillin	100	10	DBD with falling liquid film	2	0.115 × 10^1^	0.60	7213.23	[158]
Ampicillin	100	30	DBD with falling liquid film	2	3.83 × 10^−1^	1.80	2379.30	[158]
Oxacillin	100	30	DBD with falling liquid film	2	3.83 × 10^−1^	1.80	2381.12	[158]
Pentoxifylline	100	60	DBD with falling liquid film	1	4.2 × 10^−2^	16.5	2208.03	[155]
Carbamazepine	23.6	30	DBD	250–500	1.3 × 10^−1^	5.33	4.44–2.22	[161]
Clofibric acid	21.5	30	DBD	250–500	3.32 × 10^−1^	2.08	4.10–2.05	[161]
Iopromide	79.1	30	DBD	250–500	1.53 × 10^−1^	4.52	15.04–7.52	[161]

## Data Availability

Not relevant for this manuscript.
